# Differential Responses to Dietary Protein and Carbohydrate Ratio on Gut Microbiome in Obese vs. Lean Cats

**DOI:** 10.3389/fmicb.2020.591462

**Published:** 2020-10-16

**Authors:** Qinghong Li, Yuanlong Pan

**Affiliations:** Nestlé Purina Research, St. Louis, MO, United States

**Keywords:** cat, microbiome, protein, carbohydrate, obesity, diet, feline

## Abstract

More than 60% of domestic cats in the United States are either overweight or obese (OW). High-protein low-carbohydrate (HPLC) diets have been recommended for weight management for humans and pets. Gut microbes can influence the host’s health and metabolism. Less is known about feline gut microbiomes compared to other species. Thirty-nine lean (LN) and OW domestic short-haired cats (median age, 7.2 years) with median body fat of 15.8 and 32.5%, respectively, were enrolled in a two-phase study. All cats were fed the control diet (CON) with 32.4% protein and 32.3% carbohydrate for 8 weeks followed by another 8 weeks of intervention where half of the cats continued the CON diet while the other half were switched to a HPLC diet with 51.4% protein and 11.6% carbohydrate. The goal was to understand how the HPLC diet influenced gut microbiota in obese vs. lean cats. The 16S rRNA gene profiling study revealed a significant impact on gut microbiome by dietary protein and carbohydrate ratio. The effect was more pronounced in OW cats than LN cats. While no microbial taxon was different between groups in LN cats, compositional changes occurred at different taxonomical ranks in OW cats. At the phylum level, *Fusobacteria* became more abundant in HPLC-fed cats than in CON-fed cats. At the genus level, five short-chain fatty acid (SCFA) producers had altered compositions in response to the diets: *Faecalibacterium* and *Fusobacterium* are more abundant in HPLC-fed cats while the abundances of *Megasphaera*, *Bifidobacterium*, and *Veillonella* increased in CON-fed cats. Predicted microbial gene networks showed changes in energy metabolism and one-carbon metabolism pathways. Our study demonstrated differential responses to HPLC diet between obese vs. lean cats and opportunities to explore these SCFA-producers for weight management in cats.

## Introduction

Pet obesity has increased in the past two decades, and it currently is reported that more than 60% of pet cats are overweight or obese (OW)^1^. Comorbidities associated with excessive body weight include diabetes mellitus, cardiovascular diseases, musculoskeletal disorders, and many others. Thus, feline obesity poses not only health concerns for the cats, but also economical and emotional issues for the owners. Obesity can be attributed to a combination of causes including increased energy intake, reduced energy expenditure and more efficient absorption in dietary nutrients, which has been associated with changes in microflora in the gastrointestinal (GI) tract of hosts (Ley et al., 2005; Turnbaugh et al., 2006). The GI tract is home of trillions of microorganisms that ferment and utilize unabsorbed dietary components (Sonnenburg et al., 2005). Some of the bacteria in the intestine and colon metabolize dietary carbohydrates to produce short-chain fatty acids (SCFAs) which are precursors for energy production (McNeil, 1984). In humans, an estimated 5–10% of absorbed energy comes from the colon (Bingham et al., 1982; McNeil et al., 1982). GI microbes in obese individuals are thought to be more efficient at extracting energy than those in lean individuals (Backhed et al., 2004; Turnbaugh et al., 2006). The ratio of *Firmicutes* to *Bacteroidetes* in the gut has been associated with obesity in dogs, mice, and humans (Ley et al., 2005, 2006; Ley, 2010; Li et al., 2017; Coelho et al., 2018). Potential mechanisms through which gut microbes contribute to host obesity include extraction of extra dietary energy, increased lipogenesis and accumulation of triglycerides in adipocytes (Benahmed et al., 2020).

The concept of using high-protein low-carbohydrate (HPLC) diets for weight loss has been known for many years (Riegler, 1976). Potential benefits of HPLC diets, including increased satiety, reduced hunger, loss of body fat, and retention of lean body mass, were reported in humans, rodents, and dogs (Bierer and Bui, 2004; Laflamme and Hannah, 2005; Kinzig et al., 2007; Kushner and Doerfler, 2008). HPLC diets have been shown to alter the gut microbiome in kittens (Hooda et al., 2013). In dogs, a HPLC diet altered the composition and function of the gut microbiota differentially based on body condition, with greater changes observed in OW dogs (Li et al., 2017; Coelho et al., 2018). However, studies of the gut microbiome of adults cats as affected by HPLC diets or by body composition are limited. In this study, we examined the effect of dietary protein and carbohydrate on gut microbiome in adult cats with and without excessive body fat.

The goal of this study was to understand how gut microbiome changes in response to a HPLC diet vs. a control diet (CON) diet in adult cats. Based on the previous studies in dogs and kittens, we hypothesized that there would be a significant shift in microbiota between HPLC and CON diets, and that effect would be greater in OW cats than cats with lean and healthy body condition (LN).

## Materials and Methods

### Animals and Study Design

The study protocol was approved by the Institutional Animal Care and Use Committee of the Nestlé Purina PetCare Company. Twenty OW domestic short-haired neutered male or female cats and 19 sex-, breed-, and age-matched LN cats were selected for a two-phase 16-week study. Cats with a body condition score (BCS) between 4 and 5 in the 9-point scale system (Laflamme, 1997) were considered for the LN group, while cats with BCS 7–9 were considered for the OW group. Body fat was determined by quantitative magnetic resonance (QMR) analysis using EchoMRI-Infants QMR Analyzer (Echo Medical Systems) to confirm body composition prior to randomization (T0): cats with ≤20% body fat were considered LN while those with ≥25% body fat were considered OW. Cats in each weight group were randomly assigned to two diet treatment groups balanced for sex, age, and body fat: the CON and the HPLC diet as the test diet (Table 1). During the baseline feeding phase, all cats were fed the CON diet for 8 weeks during which each cat’s maintenance energy requirement (MER) was individually determined based on intake needed to maintain their body weight. Initially, MER (kilocalories) was estimated as 60×*bodyweight*(*kg*) with a weekly adjustment made if body weight increased or decreased by more than 5% of their initial body weight. In the intervention phase, cats were switched to their assigned diet and fed to maintain body weight for another 8 weeks. Fecal samples were collected, and body fat percentages were measured using QMR after the baseline (T1) and intervention (T2) period.

**TABLE 1:** Physical characteristics of the cats.

	**Lean and normal (LN)**			**Overweight or obese (OW)**		
	**HPLC**	**CON**	***P*-value**	**HPLC**	**CON**	***P*-value**
*N*	10	9		10	10	
Sex						
Female	5	4		4	4	
Male	5	5		6	6	
Age	6.75 ± 4.49	6.49 ± 3.53	0.89	7.15 ± 2.50	7.08 ± 2.23	0.95
Body weight (kg)	4.03 ± 0.78	4.19 ± 0.69	0.63	5.85 ± 1.01	5.97 ± 0.82	0.77
Body condition score	5.0 ± 0.0	5.0 ± 0.0	n/a	7.3 ± 0.7	7.4 ± 0.5	0.71
Body fat (%)^a^						
T0	14.7 ± 3.7	14.6 ± 4.5	0.94	32.3 ± 5.4	33.9 ± 3.3	0.44
T1	14.4 ± 4.0	14.9 ± 3.9	0.77	31.4 ± 5.0	32.7 ± 4.0	0.52
T2	15.7 ± 3.0	16.7 ± 4.1	0.58	32.5 ± 5.4	33.7 ± 4.0	0.58
*P*-value (ANOVA)^b^	0.73	0.53		0.89	0.75	

*^*a*^Body fat is measured by quantitative magnetic resonance (QMR) before and after baseline feeding (T0 and T1, respectively), and after intervention (T2). ^*b*^Difference in body fat within each body type by diet group. Student’s *t*-test is performed unless indicated otherwise. HPLC, high-protein low-carbohydrate diet; CON, control diet; na, not applicable. Continuous variables are expressed as mean ± standard error.*

Cats were individually housed with access to a group room with natural and supplemental lighting on a 12-h cycle. All cats received regular exercise in the activity room with other cats, routine grooming, and regular socialization by caretakers.

### Diets

The study diets were formulated to be isocaloric, and to meet or exceed the maintenance nutrient requirement based on the guidelines of the Association of American Feed Control Officials. Both diets contained animal protein as a primary protein source. The protein level in the HPLC diet was adjusted by replacing grains with plant protein. Details in nutritional compositions of the diets are described in Table 2.

**TABLE 2:** Nutrition compositions in HPLC and CON diets.

**Content (%)**	**HPLC**	**CON**
Moisture	7.82	7.98
Protein	51.35	32.43
Carbohydrate	11.60	32.30
Fat	14.60	14.30
Total dietary fiber	12.70	11.20
Soluble dietary fiber	1.18	0.79
Insoluble dietary fiber	11.50	10.40
Ash	10.3	8.80
Calculated ME (kcal/g)	3.45	3.48

*HPLC, high-protein low-carbohydrate diet; CON, control diet; ME, metabolizable energy.*

### The 16S rRNA Gene Sequencing of the V3–V4 Region

Fresh fecal samples were collected within 15 min of defecation at T1 and T2 and were immediately frozen and stored at −80°C until use. Fecal DNA extraction was performed using PowerFecal DNA isolation kit (Mo Bio Laboratories) and quantified by Quant-It Pico Green (Thermo Fisher Scientific) according to manufacturer’s protocols. The 16S rRNA gene library was constructed according to Illumina’s 16S metagenomic sequencing library preparation guide. Sequencing was performed in an Illumina MiSeq machine with 500 cycles according to the previously described procedures (Li et al., 2017).

### Bioinformatics Analysis

Each paired-end reads were merged and assembled into a single read using the software PEAR with default settings (Zhang et al., 2014), resulting in an average of 99.4% assembly. Unassembled reads and those with less than 350 or greater than 475 nucleotides were discarded. Chimeric reads were detected using UCHIME (Edgar et al., 2011) and discarded. Reads were de-replicated, sorted and clustered into operational taxonomic units (OTUs) based on minimal 97% identity using the UPARSE-OTU clustering algorithm and the greengenes database (v. 13.8) (Edgar, 2010, 2013). Taxonomy assignment was performed using the k-mer-based K-nearest neighbor search algorithm implemented in Mothur (version 1.39.5) (Schloss et al., 2009) by searching the reference sequence file from the greengenes database (Quast et al., 2013). Sequence alignment was performed using PyNAST (Caporaso et al., 2010a). Phylogenetic tree was built from the aligned sequences using FastTree (Price et al., 2009, 2010). Microbial compositional data in the OTU table was normalized using total sum scaling where relative abundance was calculated for each microbial taxa. Low abundance taxa with less than 0.01% in relative abundance in all samples were removed. Faith’s phylogenetic diversity (PD), Shannon diversity, and observed species indexes were calculated using the QIIME script “alpha_rarefaction.” Beta diversity on Bray–Curtis, weighted and unweighted UniFrac dissimilarity matrix was calculated using “beta_diversity_through_plots” (Lozupone et al., 2011). Rarefactions on the OTU table were performed to the maximal depth of 20,000 reads. QIIME (version 1.9.1) functions were called for making taxonomy assignment, OTU table and phylogenetic tree (Caporaso et al., 2010b).

### Statistical Analysis

ANOVA analysis followed by Tukey’s *post hoc* test was performed to compare alpha diversity between groups. Permutational multivariate analysis of variance (PERMANOVA) was performed to compare beta diversity (Oksanen et al., 2019). Student’s *t*-test was performed to test the null hypothesis that the means of the first or second principal component (PC), PC1 or PC2, between groups were equal. The distance from T1 and T2 in each cat was calculated using the unweighted UniFrac dissimilarity matrix and compared across four body condition-diet groups using ANOVA followed by Tukey’s *post hoc* test. To identify significant taxa between groups, only taxa with at least 50% of non-zero values in at least one group were considered and non-parametric Kruskal–Wallis test was performed followed by Dunn’s multiple comparison with Benjamini–Hochberg adjustment. Multivariate analysis by linear models (MaAsLin) was performed to find associations between clinical variables and microbial features (Morgan et al., 2012). Linear discriminant analysis (LDA) effect size (LEfSe) (Segata et al., 2011) and Random Forest machine learning with 500 trees were performed to select microbial markers that contributed most to the separation between groups using the MicrobiomeAnalyst web portal (Dhariwal et al., 2017). To evaluate functional changes in gut microbial metagenomes due to diet intervention, phylogenetic investigation of communities by reconstruction of unobserved states (PICRUSt) (Langille et al., 2013) was performed on the compositional data to estimate Kyoto Encyclopedia of Genes and Genomes (KEGG) orthologs and pathways, which were subject to LEfSe analysis. P values were adjusted to control false discovery rate (FDR) (Benjamini and Hochberg, 1995). Statistical computing was performed in R version 3.5.2. (R Core Team, 2015).

## Results

There were 10 males and 9 females in the LN group and 12 males and 8 females in the OW group (Table 1). The mean ages were 6.6 ± 0.9 (standard error) for LN cats and 7.1 ± 0.5 for OW cats (*P* = 0.64) while mean BCS for LN and OW cats were 5.0 ± 0.0 and 7.4 ± 0.13 (*P* < 0.01), respectively. At T0, the median body fat percentages were 14.0% (range, 6.9–17.3%) for LN males, 15.4% (range, 5.7–20.0%) for LN females, 32.0% (range, 26.4–37.0%) for OW males, and 34.8% (range, 28.8–45.8%) for OW females. At T2, one male and one female of the LN group had exceeded 20% body fat: means were 15.2% (10.5, 21.8%) for LN males, 17.1% (9.6, 20.8%) for LN females, 30.8% (26.2, 34.6%) for OW males, and 36.4% (29.9, 43.8%) for OW females. There was no significant change in body fat among T0, T1, and T2 (P_ANOVA_ > 0.05 in all cases, Table 1). All cats were healthy throughout the study except that one HPLC-fed LN cat (LN-HPLC) who developed a health issue unrelated to the diet after T1 and whose T2 sample was not collected.

A total of 4,809,360 paired-end sequences were generated from 77 fecal samples, with a median 61,903 sequences per sample (range, 31,538–82,908). After assembly and quality trimming, the median was 48,352 sequences (range, 24,445–67,643) per sample (Supplementary Table 1). The median length of the assembled sequences was 440 nucleotides. A operational taxonomic units (OTUs) table with 77 samples and 1,635 was constructed.

### Dietary Effects on Gut Microbial Diversities

**FIGURE 1:**
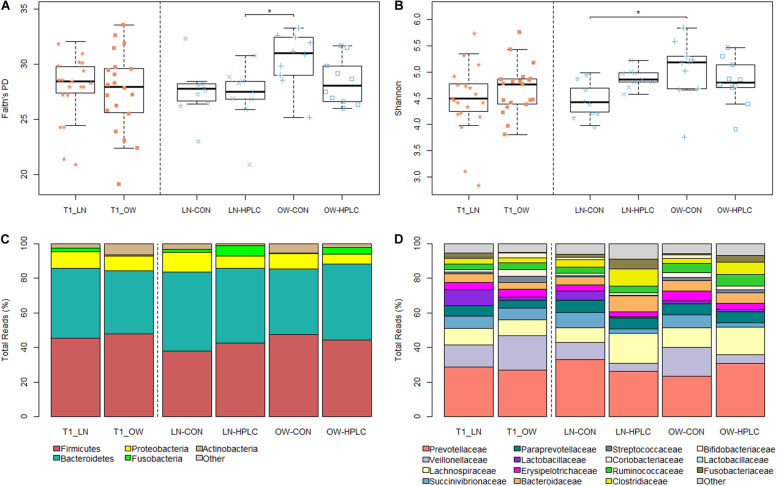
Faith’s PD **(A)** and Shannon diversity **(B)** indexes after baseline feeding (T1, orange) and intervention (T2, blue). Relative abundances of gut microbiota in the phylum level **(C)** and family level **(D)**. LN, lean cats; OW, overweight or obese cats; HPLC, high-protein, low-carbohydrate diet; CON, control diet. Adjusted *P < 0.05.

To assess richness and evenness in gut microbial communities in each cat, Faith’s PD, Shannon diversity and Observed Species indexes were calculated (Supplementary Table 2). Although no difference was found between OW and LN cats (*P* > 0.05) at T1 after all cats were fed the same CON diet, a significant difference was found at T2 (*P* < 0.05 for both Faith’s PD and Shannon indexes, Figures 1A,B). Tukey’s *post hoc* test showed significant differences in Faith’s PD between LN-HPLC cats and OW-CON cats (mean = 27.0 and 30.5, respectively, *P* = 0.019) and in Shannon diversity between LN-CON cats and OW-CON cats (mean = 4.46 and 5.45, respectively, *P* = 0.022). A similar trend was found in Observed Species index (*P*_ANOVA_ = 0.057).

**FIGURE 2:**
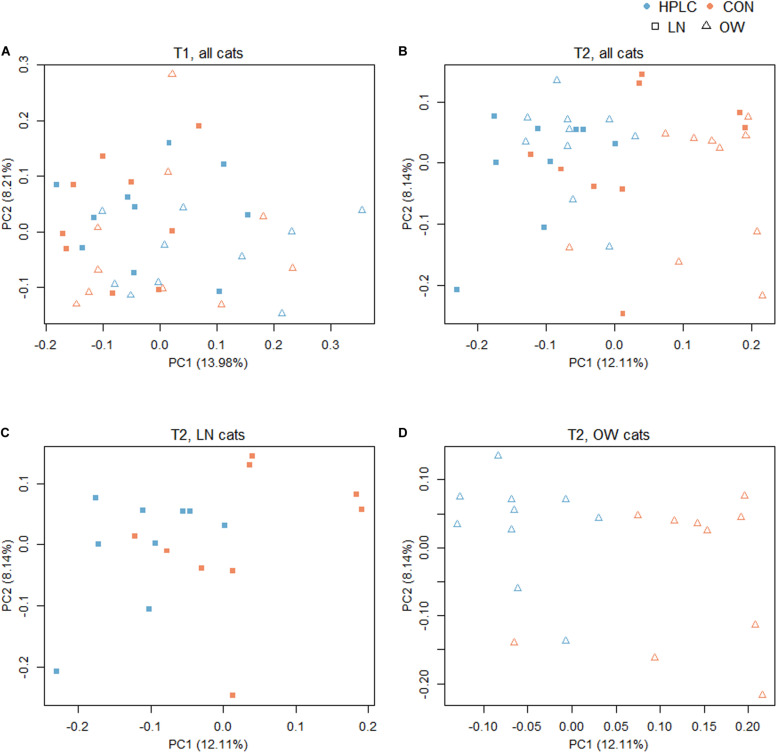
Principal coordinates analysis based on unweighted UniFrac distance metric with **(A)** all cats at T1, **(B)** all cats at T2, **(C)** LN cats at T2, and **(D)** OW cats at T2. Squares represent LN cats; triangles represent OW cats. Blue and orange represent HPLC and CON diets respectively. The percentages of data variation explained by the first two principal coordinates are indicated on the *x* and *y* axes. LN, lean cats; OW, overweight or obese cats; HPLC, high-protein, low-carbohydrate diet; CON, control diet.

To explore the effects of diet, sex, and body condition on gut microbial composition, beta diversity indexes based on Bray–Curtis, weighted and unweighted UniFrac metrics were calculated (Supplementary Table 2) (Lozupone and Knight, 2015). At T1, while no difference was found in fecal microbiomes in the principal coordinate analysis (PCoA) analysis due to sex or prospective diet groups (*P* > 0.10 in both cases, Figure 2A and Supplementary Figure 1A), a significant difference was found between OW and LN cats (*P* = 0.047, unweighted UniFrac, Supplementary Figure 1B). No difference was found between the prospective diet groups in either LN or OW cats (Supplementary Figures 1C,D).

**FIGURE 3:**
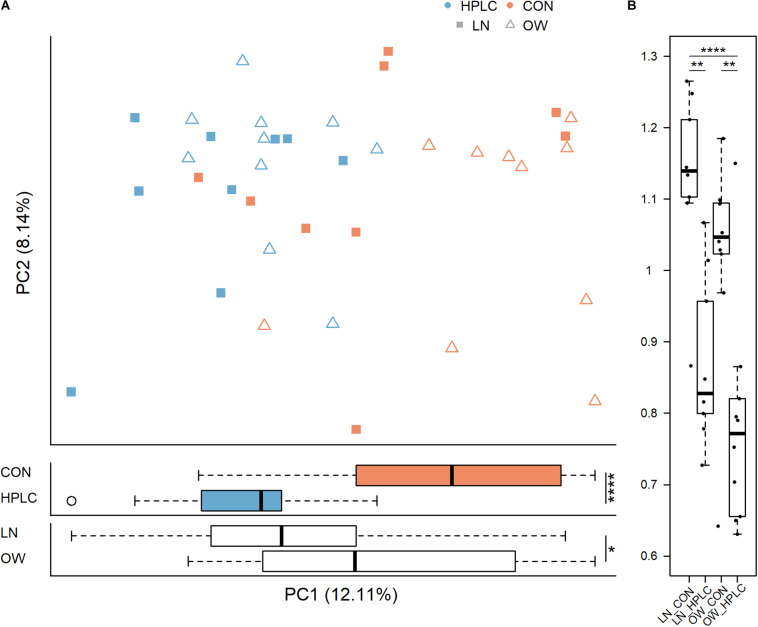
Principal coordinate analysis based on **(A)** unweighted UniFrac distance metric (top panel) and sample distribution along PC1 by diet (middle panel) and by body condition (bottom panel). **(B)** Differences in unweighted UniFrac distance from T1 to T2 for each cat are calculated and plotted for the four groups. LN, lean cats; OW, overweight or obese cats; HPLC, high-protein, low-carbohydrate diet; CON, control diet. *P*-values from student’s *t*-test **(A)** and Tukey’s post hoc test **(B)**: *P < 0.05, **P < 0.01, ***P < 0.001, ****P < 0.0001.

The dietary intervention had a significant impact on fecal microbial communities (Figure 2B and Supplementary Table 2). Significant changes were observed among the four body condition-diet groups, OW-HPLC, OW-CON, LN-HPLC, and LN-CON, using the unweighted UniFrac distance, (*P*_PERMANOVA_ = 1.0e-06). Dietary effect was greater in OW cats than in LN cats (*P*_PERMANOVA_ = 2.9e-05, *P*_PERMANOVA_ = 0.008, respectively, Figures 2C,D). The distribution of samples along the first and second principal coordinates, PC1 and PC2, was also analyzed. A significant difference along PC1 was found due to diet or body condition (*P* = 2.8e-06, *P* = 0.046, respectively, Figure 3A). No difference was found on PC2 (*P* > 0.05 in both cases). Significant changes were observed on the distance from T1 to T2 based on unweighted UniFrac dissimilarity matrix (*P* = 8.9e-06, Figure 3B).

Multivariate linear modeling was performed to assess any association between microbial abundance and clinical variables such as age, sex, body weight, BCS, and body fat. No significant association was found.

### Gut Microbiota Between OW vs. LN Cats

No taxonomic change was found at T1 after adjusting for multiple testing errors (FDR > 0.05 in all cases, Supplementary Table 3). Two phyla showed opposite trends: *Fusobacteria* was more abundant in LN cats while *Actinobacteria* was enriched in OW cats. But the differences did not reach statistical significance (*P* = 0.057 and 0.054, respectively, Figure 1C). Similar trends were found in the families of *Fusobacteriaceae* and *Coriobacteriaceae* (phylum Actinobacteria) (*P* = 0.057 and 0.030, respectively, Figure 1D). At the genus level, *Firmicutes*, *Butyrivibrio*, *Bulleidia*, *Dialister*, and *Acidaminococcus* were more abundant in OW cats while *Fusobacterium* was less abundant when compared to LN cats (*P* = 0.009, 0.010, 0.020, 0.057, 0.058, respectively, FDR > 0.05 in all cases; Supplementary Table 3).

### Dietary Intervention Changes Gut Microbiota in OW Cats

**FIGURE 4:**
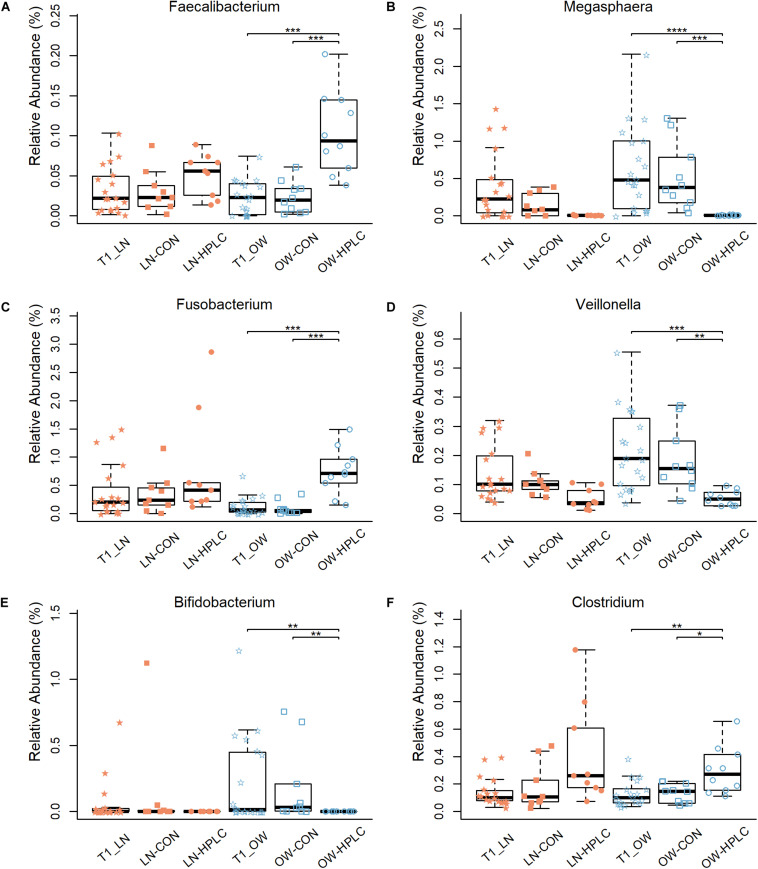
Differences in relative abundance of six bacterial genera: **(A–F)** *Faecalibacterium, Megasphaera, Fusobacterium, Veillonella, Bifidobacterium*, and *Clostridium*. Samples are from the T2 time point unless indicated otherwise. LN, lean cats; OW, overweight or obese cats; HPLC, high-protein, low-carbohydrate diet; CON, control diet. *P*-values were corrected for multiple testing (FDR): *FDR < 0.05, **FDR < 0.01, ***FDR < 0.001, ****FDR < 0.0001.

Effects of diet intervention on bacterial compositions were examined at the phylum, family, genus, and species levels in OW cats (Table 3 and Supplementary Table 4). The abundance of *Fusobacteria* was increased by more than eight fold in HPLC cats compared to CON cats (FDR = 0.001, Figure 1C). At the family level, the abundances of *Fusobacteriaceae, Clostridiaceae, Lachnospiraceae, Ruminococcaceae, Mogibacteriaceae*, and *Peptococcaceae* were increased while those of *Veillonellaceae, Bifidobacteriaceae, Porphyromonadaceae*, and *Rikenellaceae* were decreased in HPLC vs. CON cats (FDR < 0.05 in all cases, Figure 1D and Supplementary Figure 2). In addition, the abundances of five genera shifted in OW cats: increases in *Faecalibacterium* and *Fusobacterium* but decreases in *Megasphaera*, *Veillonella*, and *Bifidobacerium* were found in HPLC-fed cats when compared to CON-fed cats (FDR < 0.05 in all cases, Figure 4 and Table 3). *Clostridium* showed an increase in CON-fed vs. HPLC-fed cats. But the change did not reach statistical significance after adjusting for multiple testing (P = 0.007, FDR = 0.057).

**TABLE 3:** Differential taxa between diet groups in obese or overweight (OW) cats.

**Rank**	**Taxonomy**	***P* (KW)**	**FDR**	***P* (T1 vs. T2-CON)**	***P* (T1 vs. T2-HPLC)**	***P* (T2-CON vs. T2-HPLC)**	**FC (HPLC/CON)**
Phylum	Fusobacteria	0	0.001	0.651	0	0	8.1
Family	Fusobacteriaceae	0	0.004	0.651	0	0	8.1
Family	Veillonellaceae	0	0.004	0.691	0	0.003	–3.4
Family	Bifidobacteriaceae	0.002	0.018	0.527	0.003	0.004	−711
Family	Clostridiaceae	0.007	0.036	0.851	0.007	0.02	2.3
Family	Lachnospiraceae	0.007	0.036	0.31	0.005	0.099	1.4
Family	Ruminococcaceae	0.008	0.036	0.174	0.006	0.196	1.4
Family	Mogibacteriaceae	0.011	0.037	0.048	0.02	0.619	1.8
Family	Peptococcaceae	0.01	0.037	0.171	0.009	0.228	1.6
Family	Porphyromonadaceae	0.012	0.038	0.013	0.877	0.029	–3.5
Family	Rikenellaceae	0.015	0.041	0.022	0.505	0.021	–8.1
Genus	*Faecalibacterium*	0	0.002	0.868	0	0.001	4.5
Genus	*Megasphaera*	0	0.002	0.851	0	0.001	–83.4
Genus	*Fusobacterium*	0	0.003	0.651	0	0	8
Genus	*Veillonella*	0	0.004	0.691	0	0.003	–3.4
Genus	*Bifidobacterium*	0.002	0.019	0.527	0.003	0.004	−711
Species	*prausnitzii*	0	0.002	0.868	0	0.001	4.5
Species	*gnavus*	0	0.004	0.974	0	0.001	5
Species	*cylindroides*	0.001	0.011	0.892	0.002	0.003	na
Species	*dolichum*	0.003	0.022	0.091	0.043	0.002	na
Species	*ruminis*	0.006	0.031	0.374	0.004	0.068	na
Species	*plebeius*	0.01	0.042	0.596	0.008	0.048	–2.6
Species	*hiranonis*	0.012	0.043	0.487	0.009	0.073	2.1

*CON, control diet; HPLC, high-protein low-carbohydrate diet; T1, at the end of baseline feeding; T2, at the end of intervention; FDR, false discovery rate; FC, fold change; na, not applicable. Non-parametric Kruskall–Wallis (KW) test was performed among three groups of cats: T1, T2-HPLC, and T2-CON, followed by Dunn’s multiple comparison with Benjamini–Hochberg adjustment. *P*-values were adjusted to control for false discovery rate (FDR). Only significant taxa (FDR < 0.05) were shown.*

Four species, *Faecalibacterium prausnitzii*, *Ruminococcus gnavus*, *C*lostridium *hiranonis*, and *E*ubacterium *dolichum*, had increased abundances while three others, *Eubacterium cylindroides*, *Lactobacillus ruminis*, and *Bacteroides plebeius*, decreased (FDR < 0.05 in all cases, Table 3) in HPLC cats vs. CON cats.

No change in any taxonomic rank was found between diets in LN cats (FDR > 0.05, Supplementary Table 4).

### Biomarkers and Machine Learning

**FIGURE 5:**
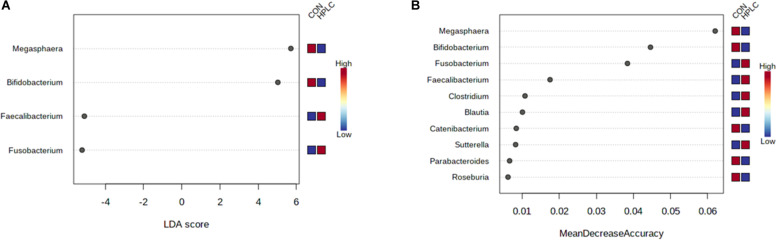
**(A)** LEfSe analysis identifies four genera significantly different between OW-HPLC vs. OW-CON cats. Selection criteria: *log*_10_⁡(*LDA*) > 3.0 and FDR < 0.05. **(B)** Top 10 genera with the most discriminant powers by Random Forest machine learning with 500 trees. LN, lean cats; OW, overweight or obese cats; HPLC, high-protein, low-carbohydrate diet; CON, control diet.

LEfSe analysis identified four genera using the selection criteria of FDR < 0.05 and *log*_10_⁡(*LDA*) > 3.0 (Supplementary Table 5). *Fusobacterium* and *Faecalibacterium* were more abundant in OW-HPLC cats while abundances of *Megasphaera* and *Bifidobacterium* were decreased (Figure 5A). No genus had a significant change between diets in LN cats using the same selection criteria.

Random Forest machine learning selected top 10 genera including *Fusobacterium, Faecalibacterium, Megasphaera, Bifidobacterium*, *Roseburia*, *Sutterella*, *Clostridium*, *Catenibacterium*, *Blautia*, and *Parabacteroides* (Figure 5B). The four genera with the greatest predictive power belonged to three phyla: *Megasphaera* and *Faecalibacterium* (*Firmicutes*), *Fusobacterium* (*Fusobacteria*), and *Bifidobacterium* (*Actinobacteria*). The resulting random forest classifier achieved an out-of-bag (OOB) error rate of 5% (Supplementary Figure 3).

### Predicted Metagenomic Functions

LEfSe analysis identified nine KEGG pathways in OW cats using the selection criteria of *P* < 0.01 and *log*_10_⁡(*LDA*) > 2.5 (Supplementary Table 6). No pathway was identified in LN cats. These pathways, all of which were enriched in CON-fed OW cats, included pathways involved in energy metabolism (glycolysis and gluconeogenesis, pyruvate metabolism, propianoate metabolism, fatty acid metabolism, and TCA cycle), and folate biosynthesis and one-carbon metabolic (one-carbon pool by folate) pathways. In addition, peptidase, tryptophan metabolism, and lysine degradation pathways were also identified as different between diets in OW cats.

## Discussion

High-protein low-carbohydrate diets have been suggested as a tool for body weight management for decades with potential benefits including increased satiety, reduced hunger, and preservation of lean body mass during weight reduction in humans and animals (Riegler, 1976; Nobels et al., 1989; Diez et al., 2002; Bierer and Bui, 2004; Laflamme and Hannah, 2005; Kushner and Doerfler, 2008). The GI microbiota plays an important role in utilizing unabsorbed food components and extracting energy from food. In this study, we examined differential responses to the HPLC diet in gut microbiomes in obese vs. lean cats. To minimize the confounding effect of body weight changes on gut microbiota, cats were fed to maintain body weight throughout the entire study.

No difference in alpha diversity was observed between LN and OW cats fed the same CON for an 8 weeks period at T1. Similar observations were reported in obese vs. lean dogs (Handl et al., 2013; Li et al., 2017). Shannon diversity showed differences between LN and OW cats fed on the CON diet for additional 8 weeks at T2. It is thus possible that the difference between body conditions was initially masked by a confounding dietary effect which diminished at T2. Fischer et al. (2017) reported differences in Faith’s PD but not Shannon diversity between lean vs. obese cats. But no change was found before and after weight loss in obese cats. It is difficult to fully assess these results with the relatively small number of microbiome studies available in cats compared to those in humans and other mammals.

Consistent with other microbiome studies on feline feces, *Firmicutes, Bacteroidetes, Proteobacteria, Actinobacteria*, and *Fusobacteria* are the five most predominant phyla. Interestingly, *Bacteroidetes* represented less than 1% of the phyla in kitten feces but its abundance increased significantly in adult cats, presumably at the expense of *Firmicutes* and *Actinobacteria* (Hooda et al., 2013; Fischer et al., 2017). Gut microbiomes shift in response to dietary changes in humans and animals (Wu et al., 2011; David et al., 2014; Li et al., 2017; Coelho et al., 2018). While no clustering was found in the PCoA plot between the two prospectively assigned diet groups at T1, a diet effect on GI microbial compositions became apparent at T2, with greater effects in OW cats than LN cats. Our results appear to be in agreement with the kitten study where distinct clusters were formed due to changes in dietary protein and carbohydrate ratio (Hooda et al., 2013). Body condition had a significant effect based on unweighted UniFrac and Bray–Curtis distance (both *P* < 0.05) at T1, but this effect diminished at T2 (*P* > 0.05), possibly masked by diet effect. Many bacterial taxonomic changes were found in the genus level in OW cats compared with little change in LN cats. Similar findings were reported in a dog study (Li et al., 2017). More research is needed to understand the mechanism underlying the differential responses to protein/carbohydrate change between obese vs. lean pets.

In contrast to findings in humans and rodents (Ley et al., 2006; Turnbaugh et al., 2009; Ley, 2010), Fischer et al. (2017) reported significantly more *Firmicutes* but less *Bacteroidetes* in neutered lean cats vs. obese cats. However, no change in either phylum was observed between OW vs. LN cats in the current study. An increase in *Fusobacteria* abundance was found in LN cats when compared to OW cats (2.38 vs. 0.67%, respectively, *P* = 0.057) at T1, although the difference did not reach statistical significance. The abundance of *Fusobacteria* in OW cats was increased in response to HPLC diet (HPLC 3.89% vs. CON 0.48%, respectively, FDR = 0.01) at T2. Interestingly, the findings reported in the kitten study were more drastic, where *Fusobacteria* comprised more than 12% of the microbiome in kittens fed a HPLC diet compared to merely 0.1% in those fed a moderate-protein moderate-carbohydrate diet (Hooda et al., 2013).

Five SCFA-producing genera had altered abundances in OW cats after the dietary intervention: the abundances of *Megasphaera*, *Veillonella*, and *Bifidobacterium* were decreased while those of *Faecalibacterium* and *Fusobacterium* were increased in HPLC-fed vs. CON-fed OW cats. In humans, a large increase in *Megasphaera* abundance with decreases in *Bifidobacterium* and *Faecalibacterium* were reported in obese individuals vs. normal ones (Turnbaugh et al., 2006; Furet et al., 2010; Schwiertz et al., 2010; Brandt and Aroniadis, 2013; Verdam et al., 2013). The exact species of *Megasphaera* that were increased in obese people was undetermined, but was thought to be one of the SCFA producers that can ferment excess carbohydrates into SCFAs and improve energy absorption (Holt, 1994; Turnbaugh et al., 2006). Thus, a decrease in *Megasphaera* may suggest a beneficial effect of the HPLC diet for weight loss in OW cats. The *Bifidobacterium* spp. possess a large number of genes involved in carbohydrate metabolism in their genomes (Lee and O’Sullivan, 2010), thus it is not surprising that this genus became more abundant in CON group vs. HPLC group. *Faecalibacterium* with a sole known species *F. prausnitzii*, is one of the most abundant and important commensal bacteria in humans, accounting for more than 5% of total bacteria in human feces (Louis et al., 2010; Arumugam et al., 2011). It is a butyrate-producing bacterium in the phylum of *Firmicutes* with anti-inflammatory effects *in vivo* and *in vitro* (Sokol et al., 2008). It has been documented that the abundance of *F. prausnitzii* was decreased in rats (Liu et al., 2014; Mu et al., 2016), pigs (Boudry et al., 2013), and dogs (Schmidt et al., 2018) fed high protein diets. To the contrary, the HPLC diet increased *F. prausnitzii* abundances in the feces of kittens (Hooda et al., 2013) and in this study, adult cats. The cat is considered an obligate carnivore that has evolved on diets rich in protein. Thus, it is conceivable that cats, which have a higher protein requirement than humans and many other mammals, may have a different response in their gut microbiota to high protein diets. Both *Veillonella* and *Fusobacterium* are SCFA producers albeit they responded differently to dietary protein and carbohydrate change in cats. The *Veillonella* spp. cannot utilize carbohydrates, but its genome encompasses genes for a major metabolic pathway which converts lactate into acetate and propionate, a SCFA with benefits in exercise endurance and athletic performance in humans and rodents (van den Bogert et al., 2013; Scheiman et al., 2019). *Fusobacterium* spp. genomes encode enzymes in the butyrate biosynthesis pathway and produce acetate and butyrate primarily through amino acid fermentation (Vital et al., 2014). Synthesis of SCFAs can activate molecular pathways that lead to lipogenesis and triglyceride accumulation in adipocytes (Benahmed et al., 2020). Thus, it is possible that decreases in *Megasphaera*, *Veillonella*, and *Bifidobacterium* may lead to reduced lipogenesis, triglyceride accumulation, and energy absorption, while increases in *Faecalibacterium* confer anti-inflammatory and immunomodulatory benefits in HPLC-fed cats compared to CON-fed OW cats.

Phylogenetic investigation of communities by reconstruction of unobserved states analysis provides an overview of microbial gene networks in the system’s level. Nine pathways were enriched in OW-CON cats compared to OW-HPLC cats. Due to the high carbohydrate content in the CON, it is not surprising to see changes in pathways involved in energy metabolism. Gut bacteria use a specific group of enzymes called CAZymes (carbohydrate-active enzymes) to break down complex carbohydrates into simple sugars (Cantarel et al., 2012), which can be further metabolized for energy through glycolysis. Glycolysis produces pyruvate, which can be converted into carbohydrates via gluconeogenesis or fatty acids through a reaction with acetyl-CoA. In addition, pyruvate can produce energy via TCA cycle if oxygen is present or via fermentation without oxygen. Gut bacteria synthesize folic acid *de novo* providing important dietary source of folates for animals. Besides being important one-carbon donors or acceptors, folic acid mediate the important one-carbon metabolism which supports multiple physiological processes including biosynthesis, amino acid homeostasis, epigenetic regulation, and redox defense (Ducker and Rabinowitz, 2017). The significance of these pathways which are based on imputed metagenomes from 16S rRNA gene composition data in CON-fed OW cats should be validated by whole metagenome sequencing.

## Conclusion

In summary, our study demonstrated a strong influence of dietary protein carbohydrate ratio on gut microbiota in adult cats. There was a significant difference in alpha diversity between OW and LN cats after 16 weeks on the CON diet. The dietary effect was evidenced in the PCoA analysis. Importantly, the effect was more prominent in OW cats than LN cats. The five predominant phyla in the feces of adult cats were *Firmicutes*, *Bacteroidetes*, *Fusobacteria*, *Actinobacteria*, and *Proteobacteria*. The OW cats had a higher *Firmicutes*/*Bacteroidetes* ratio than LN cats, although the difference did not reach statistical significance. In a metagenomic analysis between obese vs. lean twins, 75% obesity-enriched genes belonged *Actinobacteria* while the other 25% came from *Firmicutes* (Turnbaugh et al., 2009). Remarkably, the OW cats had three times as many *Actinobacteria* as their LN counterparts (*P* = 0.054). In humans, *Fusobacteria* was positively associated with obesity (Andoh et al., 2016; Gao et al., 2018; Crovesy et al., 2020). To the contrary, a decrease in *Fusobacteria* was observed in OW cats vs. LN cats (*P* = 0.057). More studies are needed to confirmed the findings. However, the HPLC diet increased *Fusobacteria* abundance in OW cats to a level comparable to that in LN cats. Changes in numerous SCFA-producers and carbohydrate-fermenters were observed. While no significant taxa change was found in LN cats, five genera were changed in OW cats: *Faecalibacterium* and *Fusobacterium* were more abundant while *Megasphaera*, *Veillonella*, and *Bifidobacerium* became less abundant in HPLC-fed cats compared to CON-fed cats. On the pathway level, changes in energy and one-carbon metabolisms were noticed. Future metagenomics sequencing and analysis may provide additional resolutions in the species or even strain levels and opportunity to better understand metagenomic changes in the systems level.

## Data Availability Statement

The sequence and meta data in the study can be obtained through the accession number PRJNA661962 from the Sequence Read Archive database.

## Ethics Statement

The animal study was reviewed and approved by the Institutional Animal Care and Use Committee of the Nestlé Purina PetCare Company.

## Author Contributions

QL conceived the study, participated in the feeding study, performed data analysis and interpretation, and wrote the manuscript. YP designed the diets, performed diet analysis, participated in the feeding study, and reviewed the manuscript. Both authors contributed to the article and approved the submitted version.

## Conflict of Interest

QL and YP are current employees of the Nestlé Purina PetCare Company.

## Footnotes

1 https://petobesityprevention.org/

## Supplementary Material

Supplementary Figure 1 Principal coordinates analysis (PCoA) based on unweighted UniFrac distance metric on **(A)** sex, **(B)** body condition, and diet in **(C)** LN cats and **(D)** OW cats at T1. The percentages of data variation explained by the first two principal coordinates are indicated on the *x* and *y* axes.

Supplementary Figure 2 Differences in relative abundances of ten bacterial families. T1 samples are denoted. *P* values were corrected for multiple testing (FDR). *FDR < 0.05, **FDR < 0.01, ***FDR < 0.001.

Supplementary Figure 3 Performance of Random Forest machine learning classifier over number of trees.

Supplementary Table 1 Sequence distribution by sample, assembly, and passage rates.

Supplementary Table 2 Alpha- and beta-diversities between groups at T1 and T2. ANOVA and Tukey’s *post hoc* test were applied to compare alpha diversities while PERMANNOVA is used to compare beta diversities.

Supplementary Table 3 Changes in taxonomical abundances between OW and LN cats at T1. *P*-values from Mann–Whitney U test are obtained and adjusted for multiple testing error.

Supplementary Table 4 Comparison of the diet groups at the phylum, family, genus, and species levels in OW and LN cats using Kruskal–Wallis test followed by Dunn’s multiple comparison with Benjamini–Hochberg adjustment. *P*-values are adjusted for multiple testing error using false discovery rate (FDR).

Supplementary Table 5 LEfSe analysis on bacterial genus between OW-HPLC vs. OW-CON cats. LEfSe selection criteria: FDR < 0.05 and *log*_10_⁡(*L**D**A*) > 3.0.

Supplementary Table 6 PICRUSt-predicted KEGG metagenomic orthologous functions between OW-HPLC and OW-CON cats. KO pathways were selected using *P* < 0.05 and *log*_10_⁡(*L**D**A*) > 2.5.

## Abbreviations

CON: control diet
FDR: false discovery rate
HPLC: high-protein low-carbohydrate diet
KEGG: Kyoto Encyclopedia of Genes and Genomes
LEfSe: linear discriminant analysis effect size
LN: lean and normal weight
OTU: operational taxonomic unit
OW: overweight or obese
PCoA: principal coordinate analysis
PD: phylogenetic diversity
PICRUSt: phylogenetic investigation of communities by reconstruction of unobserved states
SCFAs: short-chain fatty acids.
