# Supplementary material for: Differential Responses to Dietary Protein and Carbohydrate Ratio on Gut Microbiome in Obese vs. Lean Cats
Source: Front Microbiol. 2020 Oct 16;11:591462. doi: 10.3389/fmicb.2020.591462 (PMC7596662; doi:10.3389/fmicb.2020.591462)
Supplement: Supplementary Figure 1 — Principal coordinates analysis (PCoA) based on unweighted UniFrac distance metric on (A) sex, (B) body condition, and diet in (C) LN cats and (D) OW cats at T1. The percentages of data variation explained by the first two principal coordinates are indicated on the x and y axes. [file Data_Sheet_2.pdf]

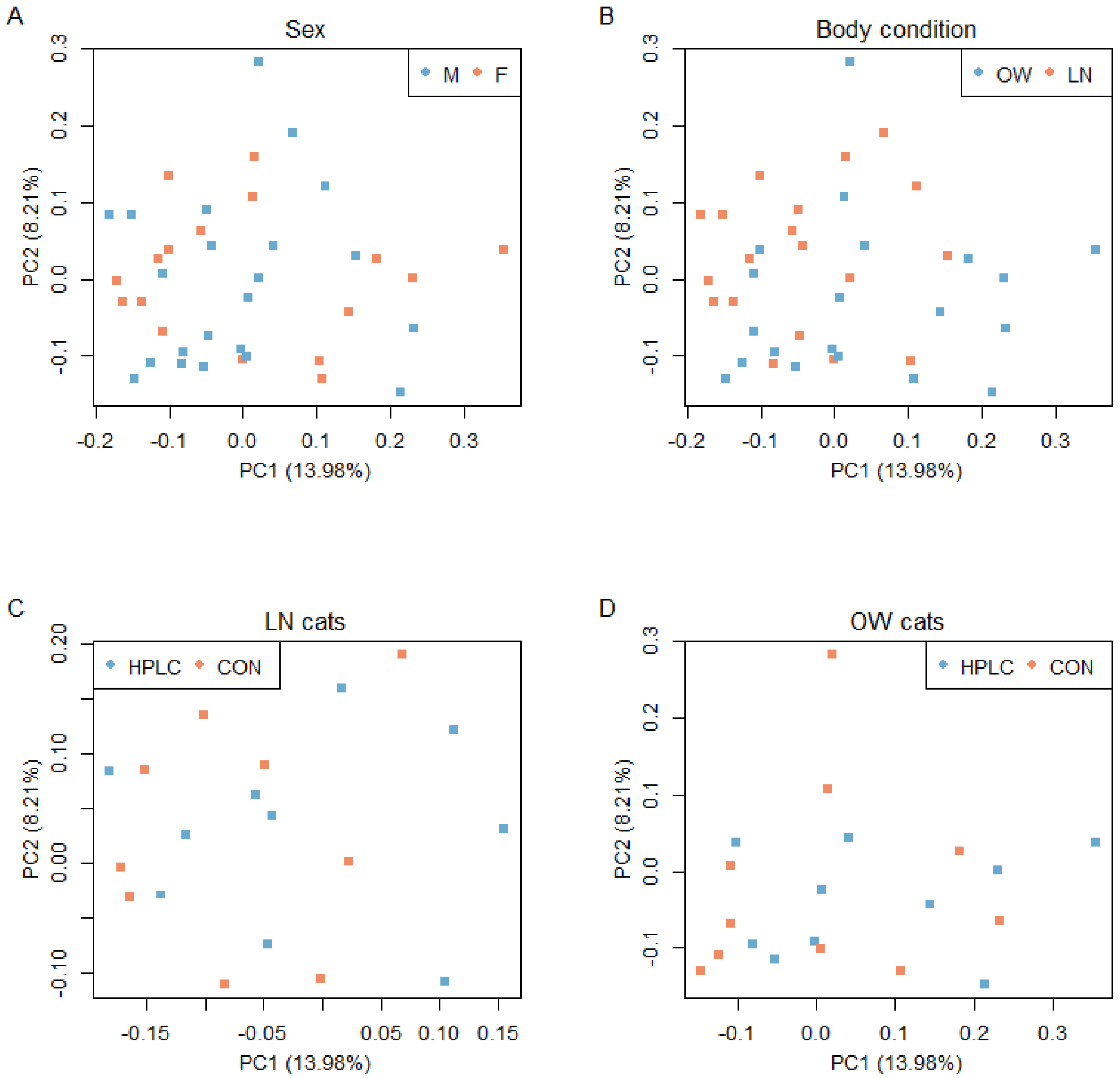

Fig S1. Principal coordinates analysis (PCoA) based on unweighted UniFrac distance on (A) sex, (B) body condition, and diet in (C) LN cats and (D) OW cats at T1. The percentages of data variation explained by the first two principal coordinates are indicated on the x and y axes.

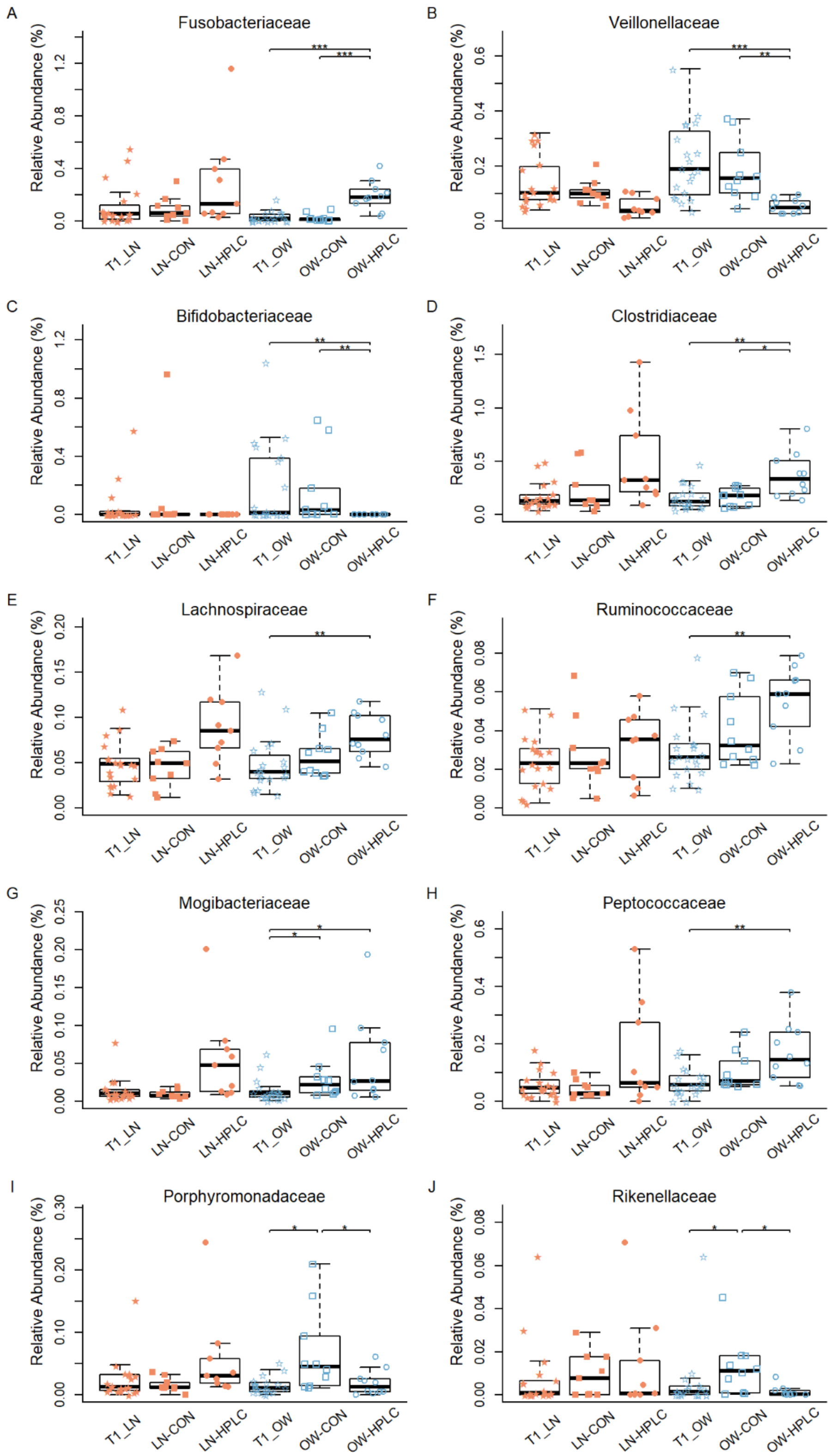

Fig S2. Differences in relative abundances of ten bacterial families. T1 samples are denoted. The P values were corrected for multiple testing (FDR).
*FDR < 0.05, **FDR < 0.01, ***FDR < 0.001.

A

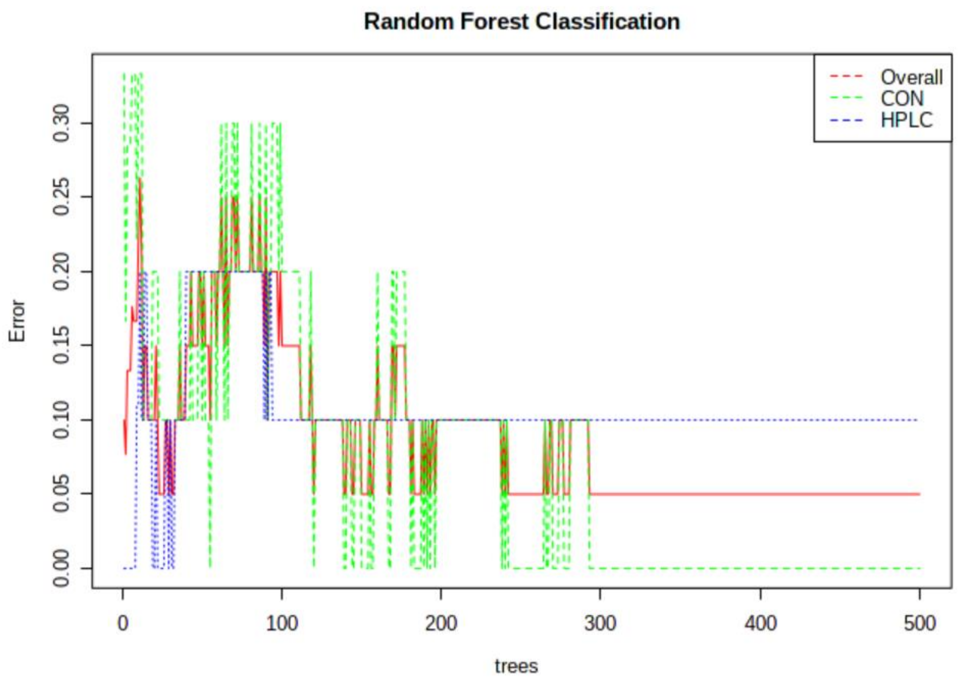

B

The OOB error is 0.05

	CON	HPLC	class.error
CON	10	0	0.0
HPLC	1	9	0.1

Fig S3. (A) Performance of Random Forest machine learning classifier over number of trees and (B) the out-of-bag (OOB) error rate.
