# Supplementary material for: Differential Responses to Dietary Protein and Carbohydrate Ratio on Gut Microbiome in Obese vs. Lean Cats
Source: Front Microbiol. 2020 Oct 16;11:591462. doi: 10.3389/fmicb.2020.591462 (PMC7596662; doi:10.3389/fmicb.2020.591462)
Supplement: Supplementary Table 1 — Sequence distribution by sample, assembly, and passage rates. [file Data_Sheet_1.pdf]

**Table S1.** Sequence (read) distribution by sample, assembly and passage rates.

| SampleID | Raw_reads | Assembled_reads | Assembly_rate | Trimmed_reads | Pass_rate |
|---|---|---|---|---|---|
| 1 | 165816 | 82251 | 99.21 | 67643 | 82.24 |
| 2 | 149422 | 74237 | 99.37 | 58466 | 78.76 |
| 3 | 133368 | 66152 | 99.2 | 49744 | 75.2 |
| 4 | 137120 | 68087 | 99.31 | 49906 | 73.3 |
| 5 | 115264 | 57143 | 99.15 | 43911 | 76.84 |
| 6 | 118418 | 58818 | 99.34 | 46595 | 79.22 |
| 7 | 115304 | 57322 | 99.43 | 44165 | 77.05 |
| 8 | 136032 | 67592 | 99.38 | 55203 | 81.67 |
| 9 | 148552 | 73716 | 99.25 | 59445 | 80.64 |
| 10 | 133458 | 66268 | 99.31 | 51907 | 78.33 |
| 11 | 123806 | 61498 | 99.35 | 48315 | 78.56 |
| 12 | 132764 | 65935 | 99.33 | 50087 | 75.96 |
| 13 | 119784 | 59600 | 99.51 | 45569 | 76.46 |
| 14 | 128342 | 63754 | 99.35 | 51414 | 80.64 |
| 15 | 130536 | 64886 | 99.41 | 48918 | 75.39 |
| 16 | 131726 | 65411 | 99.31 | 52950 | 80.95 |
| 17 | 120224 | 59679 | 99.28 | 45654 | 76.5 |
| 18 | 120388 | 59829 | 99.39 | 44075 | 73.67 |
| 19 | 122154 | 60369 | 98.84 | 45438 | 75.27 |
| 20 | 122616 | 60871 | 99.29 | 45796 | 75.23 |
| 21 | 117830 | 58350 | 99.04 | 44187 | 75.73 |
| 22 | 137416 | 68182 | 99.23 | 51444 | 75.45 |
| 23 | 119902 | 59563 | 99.35 | 46244 | 77.64 |
| 24 | 111434 | 55310 | 99.27 | 43702 | 79.01 |
| 25 | 138372 | 68803 | 99.45 | 57644 | 83.78 |
| 26 | 117872 | 58601 | 99.43 | 44890 | 76.6 |
| 27 | 103938 | 51676 | 99.44 | 42767 | 82.76 |
| 28 | 134356 | 66881 | 99.56 | 54452 | 81.42 |
| 29 | 63076 | 31274 | 99.16 | 24445 | 78.16 |
| 30 | 122230 | 60774 | 99.44 | 50975 | 83.88 |
| 31 | 108338 | 53913 | 99.53 | 45922 | 85.18 |
| 32 | 109760 | 54613 | 99.51 | 45637 | 83.56 |
| 33 | 126384 | 62859 | 99.47 | 50649 | 80.58 |
| 34 | 130694 | 65011 | 99.49 | 50151 | 77.14 |
| 35 | 142546 | 70962 | 99.56 | 53236 | 75.02 |
| 36 | 148056 | 73662 | 99.51 | 56081 | 76.13 |
| 37 | 135748 | 67591 | 99.58 | 52739 | 78.03 |
| 38 | 145434 | 72377 | 99.53 | 57241 | 79.09 |
| 39 | 124044 | 61696 | 99.47 | 47864 | 77.58 |
| 40 | 162774 | 80937 | 99.45 | 65159 | 80.51 |

| | | | | | |
|---|---|---|---|---|---|
| 41 | 136632 | 67979 | 99.51 | 55012 | 80.92 |
| 42 | 120312 | 59853 | 99.5 | 43697 | 73.01 |
| 43 | 110718 | 55087 | 99.51 | 44910 | 81.53 |
| 44 | 127598 | 63527 | 99.57 | 48609 | 76.52 |
| 45 | 106210 | 52752 | 99.34 | 41624 | 78.91 |
| 46 | 114444 | 56765 | 99.2 | 42875 | 75.53 |
| 47 | 121858 | 60607 | 99.47 | 50681 | 83.62 |
| 48 | 128324 | 63891 | 99.58 | 54635 | 85.51 |
| 49 | 137700 | 68026 | 98.8 | 51923 | 76.33 |
| 50 | 137832 | 68157 | 98.9 | 52636 | 77.23 |
| 51 | 119116 | 59169 | 99.35 | 46045 | 77.82 |
| 52 | 123678 | 61399 | 99.29 | 47632 | 77.58 |
| 53 | 108572 | 53952 | 99.38 | 42307 | 78.42 |
| 54 | 125828 | 62455 | 99.27 | 49400 | 79.1 |
| 55 | 127710 | 63436 | 99.34 | 48809 | 76.94 |
| 56 | 148098 | 73330 | 99.03 | 57767 | 78.78 |
| 57 | 116752 | 57979 | 99.32 | 44658 | 77.02 |
| 58 | 122696 | 60931 | 99.32 | 46560 | 76.41 |
| 59 | 117342 | 58392 | 99.52 | 45350 | 77.66 |
| 60 | 128878 | 64060 | 99.41 | 51856 | 80.95 |
| 61 | 101754 | 50534 | 99.33 | 40183 | 79.52 |
| 62 | 127646 | 63439 | 99.4 | 50602 | 79.76 |
| 63 | 123006 | 61087 | 99.32 | 49128 | 80.42 |
| 64 | 122656 | 60972 | 99.42 | 52135 | 85.51 |
| 65 | 124094 | 61738 | 99.5 | 47626 | 77.14 |
| 66 | 117246 | 58335 | 99.51 | 46238 | 79.26 |
| 67 | 117308 | 58393 | 99.56 | 46061 | 78.88 |
| 68 | 102914 | 51132 | 99.37 | 41506 | 81.17 |
| 69 | 118920 | 59175 | 99.52 | 46295 | 78.23 |
| 70 | 124992 | 62212 | 99.55 | 50761 | 81.59 |
| 71 | 137886 | 68619 | 99.53 | 52510 | 76.52 |
| 72 | 129462 | 64372 | 99.45 | 52863 | 82.12 |
| 73 | 109240 | 54294 | 99.4 | 40443 | 74.49 |
| 74 | 127058 | 63147 | 99.4 | 44730 | 70.83 |
| 75 | 125134 | 62247 | 99.49 | 48352 | 77.68 |
| 76 | 115690 | 57427 | 99.28 | 42143 | 73.39 |
| 77 | 108118 | 53741 | 99.41 | 41086 | 76.45 |

**Table S2.** Alpha- and beta-diversities between groups at T1 and T2. ANOVA and Tukey's host-hoc test were applied to compare alpha diversities while PERMANNOVA was used to compare beta diversities.

Alpha diversity at T1

| | | Mean | | | | P values (Tukey's post-hoc) | | | | | |
|---|---|---|---|---|---|---|---|---|---|---|---|
| | P_ANOVA | LN_CON | LN_HPLC | OW_CON | OW_HPLC | LN_HPLC vs LN_CON | vs LN_CON | vs LN_CON | vs LN_HPLC | vs LN_HPLC | OW_HPLC vs OW_CON |
| observed_species | 0.576124 | 275.3556 | 275.02 | 287.59 | 257.78 | 0.99999865 | 0.94246848 | 0.85018725 | 0.93328458 | 0.8472505 | 0.502829585 |
| Faith PD | 0.619678 | 28.18141 | 27.52492 | 28.57256 | 26.66411 | 0.97451804 | 0.99435712 | 0.76553474 | 0.90013285 | 0.94115941 | 0.59545379 |
| shannon | 0.592242 | 4.51008 | 4.467515 | 4.80015 | 4.553547 | 0.99856028 | 0.70463743 | 0.99846745 | 0.58714421 | 0.987533 | 0.782493781 |

Alpha diversity at T2

| | | Mean | | | | P values (Tukey's post-hoc) | | | | | |
|---|---|---|---|---|---|---|---|---|---|---|---|
| | P_ANOVA | LN_CON | LN_HPLC | OW_CON | OW_HPLC | LN_HPLC vs LN_CON | OW_CON vs LN_CON | OW_HPLC vs LN_CON | OW_CON vs LN_HPLC | OW_HPLC vs LN_HPLC | OW_HPLC vs OW_CON |
| observed_species | 0.056964 | 269.76667 | 278.33333 | 314.31 | 292.58 | 0.958124792 | 0.052867657 | 0.525866935 | 0.154696486 | 0.82722051 | 0.543807389 |
| Faith's PD | 0.020166 | 27.657003 | 27.003788 | 30.523092 | 28.636467 | 0.94207774 | 0.071762176 | 0.82150714 | 0.018519982 | 0.480459296 | 0.331037582 |
| shannon | 0.033972 | 4.4606639 | 4.8826083 | 5.0473898 | 4.8155894 | 0.162749553 | 0.022101128 | 0.271836498 | 0.827548313 | 0.985290346 | 0.608760269 |

Beta diversity (all cats)

| | T1 | | | | T2 | | | |
|---|---|---|---|---|---|---|---|---|
| | P_BC* x diet** | P_diet | P_sex | P_BC | P_BC x diet | P_diet | P_sex | P_BC |
| unweighted_unifrac | 0.358867641 | 0.4023016 | 0.24083176 | 0.04666395 | 1E-06 | 2E-06 | 0.63257137 | 0.06810193 |
| weighted_unifrac | 0.182071818 | 0.12513087 | 0.22056178 | 0.14415586 | 0.001212 | 0.00231 | 0.38853661 | 0.24081776 |
| bray_curtis | 0.069299931 | 0.10893689 | 0.11534088 | 0.04581295 | 8E-06 | 1E-06 | 0.68396532 | 0.32127768 |

* BC: body condition
** prospectively assigned diets

Beta diversity (LN cats vs. OW cats) at T2

| | LN cats | OW cats |
|---|---|---|
| | P_HPLC vs CON | P_HPLC vs CON |
| unweighted_unifrac | 0.007689992 | 2.9E-05 |
| weighted_unifrac | 0.046966953 | 0.004106996 |
| bray_curtis | 0.005172995 | 0.000238 |

**Table S3.** Changes in taxonomical abundances between OW and LN cats at T1. P values from Mann-Whitney U test were adjusted for multiple testing (q).

Phylum

| | p | q | mean_LN | mean_OW | N*_LN | N_OW |
|---|---|---|---|---|---|---|
| Actinobacteria | 0.053572 | 0.200438 | 2.202099 | 6.4972552 | 19 | 20 |
| Fusobacteria | 0.057268 | 0.200438 | 2.378472 | 0.6685192 | 19 | 20 |
| Bacteroidetes | 0.133938 | 0.247617 | 40.37001 | 36.364616 | 19 | 20 |
| Firmicutes | 0.141495 | 0.247617 | 45.46411 | 47.835808 | 19 | 20 |
| Cyanobacteria | 0.516472 | 0.694715 | 0.115475 | 0.0005882 | 2 | 4 |
| Chlamydiae | 0.59547 | 0.694715 | 0.004272 | 0.0016746 | 6 | 5 |
| Proteobacteria | 0.728381 | 0.728381 | 9.307912 | 8.4723544 | 19 | 20 |

Family

| | p | q | mean_LN | mean_OW | N*_LN | N_OW |
|---|---|---|---|---|---|---|
| Coriobacteriaceae | 0.030461 | 0.645332 | 1.419457 | 3.956077 | 19 | 20 |
| Fusobacteriaceae | 0.057268 | 0.645332 | 2.378472 | 0.668519 | 19 | 20 |
| Veillonellaceae | 0.061169 | 0.645332 | 12.83048 | 19.63308 | 19 | 20 |
| Peptostreptococcaceae | 0.078222 | 0.645332 | 0.26022 | 0.089788 | 15 | 17 |
| Bifidobacteriaceae | 0.190109 | 0.902616 | 0.778083 | 2.53982 | 16 | 16 |
| Alcaligenaceae | 0.213797 | 0.902616 | 1.231261 | 0.859086 | 19 | 20 |
| Turicibacteraceae | 0.241341 | 0.902616 | 0.347493 | 0.249257 | 13 | 11 |
| Lactobacillaceae | 0.266965 | 0.902616 | 8.919382 | 1.885892 | 16 | 20 |
| Prevotellaceae | 0.270427 | 0.902616 | 28.68811 | 27.02857 | 19 | 20 |
| Actinomycetaceae | 0.320924 | 0.902616 | 0.004559 | 0.001132 | 9 | 7 |
| Desulfovibrionaceae | 0.330298 | 0.902616 | 0.270009 | 0.157758 | 15 | 16 |
| Ruminococcaceae | 0.336307 | 0.902616 | 3.048753 | 3.788053 | 19 | 20 |
| Porphyromonadaceae | 0.365265 | 0.902616 | 0.223337 | 0.13987 | 19 | 20 |
| Pasteurellaceae | 0.382928 | 0.902616 | 0.000206 | 0.000637 | 2 | 4 |
| Peptococcaceae | 0.415146 | 0.913322 | 0.233287 | 0.267398 | 18 | 19 |
| Erysipelotrichaceae | 0.478013 | 0.913754 | 4.539173 | 4.355786 | 19 | 20 |
| Bacteroidaceae | 0.51333 | 0.913754 | 4.725245 | 4.00174 | 19 | 20 |
| Paraprevotellaceae | 0.587736 | 0.913754 | 6.219652 | 4.6775 | 19 | 20 |
| Chlamydiaceae | 0.59547 | 0.913754 | 0.004272 | 0.001675 | 6 | 5 |
| Barnesiellaceae | 0.605294 | 0.913754 | 0.000327 | 0.003506 | 1 | 2 |
| Clostridiaceae | 0.666661 | 0.913754 | 3.310129 | 3.040788 | 19 | 20 |
| Lachnospiraceae | 0.666661 | 0.913754 | 9.526278 | 9.470745 | 19 | 20 |
| Odoribacteraceae | 0.704167 | 0.913754 | 0.156524 | 0.199476 | 16 | 18 |
| Enterobacteriaceae | 0.72537 | 0.913754 | 0.286492 | 0.204475 | 18 | 18 |
| Helicobacteraceae | 0.742985 | 0.913754 | 0.325619 | 0.417693 | 13 | 14 |
| Rikenellaceae | 0.764643 | 0.913754 | 0.068812 | 0.047653 | 13 | 14 |
| S24-7 | 0.767747 | 0.913754 | 0.163296 | 0.134731 | 16 | 18 |
| Christensenellaceae | 0.775307 | 0.913754 | 0.001119 | 0.001324 | 5 | 4 |

| | | | | | | |
|---|---|---|---|---|---|---|
| Campylobacteraceae | 0.87684 | 0.945577 | 0.128707 | 0.259082 | 16 | 16 |
| Mogibacteriaceae | 0.878599 | 0.945577 | 0.162851 | 0.157663 | 19 | 20 |
| Streptococcaceae | 0.888269 | 0.945577 | 1.247319 | 3.475649 | 18 | 19 |
| Succinivibrionaceae | 0.988922 | 1 | 6.97651 | 6.555883 | 19 | 20 |
| Enterococcaceae | 1 | 1 | 0.05377 | 0.527075 | 10 | 13 |

Genus

| | p | q | mean_LN | mean_OW | N*_LN | N_OW |
|---|---|---|---|---|---|---|
| Butyrivibrio | 0.008778 | 0.333277 | 0.009522 | 0.0396838 | 8 | 15 |
| Bulleidia | 0.010751 | 0.333277 | 1.243459 | 2.214638 | 19 | 20 |
| Dialister | 0.020421 | 0.422036 | 0.946266 | 2.5946778 | 17 | 19 |
| Fusobacterium | 0.057268 | 0.598606 | 2.005411 | 0.6592821 | 19 | 20 |
| Acidaminococcus | 0.058406 | 0.598606 | 0.040177 | 0.8409013 | 10 | 13 |
| Veillonella | 0.061169 | 0.598606 | 12.83048 | 19.63308 | 19 | 20 |
| Allobaculum | 0.072875 | 0.598606 | 0.000449 | 0 | 3 | 3 |
| Mogibacterium | 0.077239 | 0.598606 | 0.00331 | 0.0075232 | 7 | 12 |
| Mitsuokella | 0.088679 | 0.610897 | 0 | 0.1399202 | 3 | 3 |
| Anaerobiospirillum | 0.118244 | 0.631025 | 2.812635 | 1.3471378 | 17 | 16 |
| Phascolarctobacterium | 0.12852 | 0.631025 | 2.229074 | 1.3500551 | 18 | 15 |
| Collinsella | 0.133938 | 0.631025 | 1.262169 | 2.1042453 | 19 | 20 |
| Megasphaera | 0.141495 | 0.631025 | 7.900844 | 12.378731 | 18 | 20 |
| Dorea | 0.149367 | 0.631025 | 0.842535 | 0.7210656 | 19 | 20 |
| Prevotella | 0.174937 | 0.631025 | 34.90234 | 31.696287 | 19 | 20 |
| Oribacterium | 0.187642 | 0.631025 | 0.064267 | 0.1233846 | 13 | 16 |
| Bifidobacterium | 0.190109 | 0.631025 | 0.77773 | 2.5396151 | 16 | 16 |
| Porphyromonas | 0.197478 | 0.631025 | 0.000414 | 0.0023948 | 4 | 8 |
| RFN20 | 0.20317 | 0.631025 | 0.003704 | 0.0013687 | 8 | 5 |
| Eubacterium | 0.203556 | 0.631025 | 1.768381 | 0.9480163 | 19 | 20 |
| Sutterella | 0.213797 | 0.631209 | 1.230577 | 0.8584507 | 19 | 20 |
| Turicibacter | 0.241341 | 0.680144 | 0.347493 | 0.2492575 | 13 | 11 |
| Lactobacillus | 0.266965 | 0.719471 | 8.91889 | 1.885892 | 16 | 20 |
| Faecalibacterium | 0.282858 | 0.719471 | 1.004731 | 0.7141506 | 19 | 19 |
| Actinomyces | 0.320924 | 0.719471 | 0.004559 | 0.0011322 | 9 | 7 |
| Epulopiscium | 0.329719 | 0.719471 | 9.96E-05 | 0 | 1 | 1 |
| Desulfovibrio | 0.330298 | 0.719471 | 0.270218 | 0.1578781 | 15 | 16 |
| Plesiomonas | 0.336048 | 0.719471 | 0.002829 | 0.0017701 | 4 | 2 |
| Holdemania | 0.336527 | 0.719471 | 0.001978 | 0.0032488 | 10 | 6 |
| Gallibacterium | 0.350456 | 0.724275 | 0.000118 | 0.0004106 | 1 | 3 |
| Parabacteroides | 0.380296 | 0.732937 | 0.222924 | 0.137475 | 19 | 20 |
| Pasteurella | 0.382928 | 0.732937 | 0.000206 | 0.000637 | 2 | 4 |
| Adlercreutzia | 0.390112 | 0.732937 | 0.009861 | 0.0094931 | 17 | 15 |
| Peptococcus | 0.415146 | 0.757031 | 0.233287 | 0.2673982 | 18 | 19 |
| Campylobacter | 0.508314 | 0.878142 | 0.454326 | 0.6767741 | 17 | 16 |

| | | | | | | |
|---|---|---|---|---|---|---|
| Bacteroides | 0.51333 | 0.878142 | 4.718239 | 3.9965898 | 19 | 20 |
| Peptostreptococcus | 0.524052 | 0.878142 | 0.003122 | 0.0011024 | 6 | 5 |
| Paraprevotella | 0.587736 | 0.906275 | 6.219652 | 4.6774997 | 19 | 20 |
| Barnesiella | 0.605294 | 0.906275 | 0.000327 | 0.0035055 | 1 | 2 |
| SMB53 | 0.614351 | 0.906275 | 0.000519 | 0.0005765 | 4 | 3 |
| Clostridium | 0.626677 | 0.906275 | 3.275098 | 3.0144179 | 19 | 20 |
| Alistipes | 0.641302 | 0.906275 | 0.0095 | 0.0147372 | 10 | 13 |
| Catenibacterium | 0.646545 | 0.906275 | 1.361523 | 1.12652 | 19 | 19 |
| Candidatus Arthromitus | 0.647748 | 0.906275 | 0.031949 | 0.0239406 | 9 | 7 |
| Escherichia | 0.683643 | 0.906275 | 0.283331 | 0.202014 | 18 | 18 |
| Coprococcus | 0.687015 | 0.906275 | 0.429081 | 0.4504238 | 19 | 19 |
| Ruminococcus | 0.687015 | 0.906275 | 1.437624 | 1.4172013 | 19 | 20 |
| Odoribacter | 0.704167 | 0.909549 | 0.156524 | 0.1994759 | 16 | 18 |
| Megamonas | 0.728381 | 0.918715 | 1.679308 | 2.2556066 | 19 | 20 |
| Helicobacter | 0.742985 | 0.918715 | 0.325619 | 0.4176925 | 13 | 14 |
| Roseburia | 0.76793 | 0.918715 | 0.309283 | 0.415571 | 18 | 18 |
| Slackia | 0.770535 | 0.918715 | 0.036411 | 0.0363152 | 19 | 20 |
| Oscillospira | 0.791872 | 0.925491 | 0.526728 | 0.6121032 | 19 | 20 |
| p-75-a5 | 0.806073 | 0.925491 | 0.002062 | 0.0017135 | 7 | 7 |
| Anaerotruncus | 0.857129 | 0.962654 | 0.003101 | 0.0028186 | 7 | 7 |
| Streptococcus | 0.888269 | 0.962654 | 1.247319 | 3.4756491 | 18 | 19 |
| Blautia | 0.900547 | 0.962654 | 3.372937 | 3.2463426 | 19 | 20 |
| Butyricicoccus | 0.900547 | 0.962654 | 0.175445 | 0.1912161 | 19 | 20 |
| Butyricimonas | 0.970686 | 1 | 0.000683 | 0.0002844 | 1 | 1 |
| Shigella | 0.970686 | 1 | 0.000125 | 0.0001097 | 1 | 1 |
| Succinivibrio | 0.988922 | 1 | 6.97651 | 6.555883 | 19 | 20 |
| Enterococcus | 1 | 1 | 0.05377 | 0.5270755 | 10 | 13 |

*Number of non-zero samples

**Table S4.** Comparison of the diet groups at the phylum, family, genus, and species levels in OW and LN cats using Kruskal-Wallis test followed by Dunn's multiple comparisons with Benjamini-Hochberg corrections. P values from Kruskal-Wallis tests were adjusted for multiple testing error using false discovery rate (FDR).

LN cats

| | | | | P values (Dunn's test) | | | Mean | | |
|---|---|---|---|---|---|---|---|---|---|
| Rank | Taxonomy | P_KW* | FDR | T1_CON vs T2_CON | T1_CON vs T2_HPLC | T2_CON vs T2_HPLC | T1_CON | T2_CON | T2_HPLC |
| Phylum | Fusobacteria | 0.172 | 0.613 | 0.939 | 0.215 | 0.208 | 2.378 | 1.874 | 5.947 |
| Phylum | Proteobacteria | 0.245 | 0.613 | 0.341 | 0.475 | 0.281 | 9.308 | 11.515 | 7.044 |
| Phylum | Actinobacteria | 0.782 | 0.935 | 1 | 0.771 | 1 | 2.202 | 2.945 | 1.221 |
| Phylum | Bacteroidetes | 0.935 | 0.935 | 1 | 1 | 0.965 | 40.37 | 45.467 | 43.033 |
| Phylum | Firmicutes | 0.677 | 0.935 | 1 | 0.95 | 0.744 | 45.464 | 37.978 | 42.6 |
| Family | Bacteroidaceae | 0.003 | 0.071 | 0.976 | 0.004 | 0.007 | 4.725 | 4.604 | 9.216 |
| Family | Mogibacteriaceae | 0.007 | 0.071 | 0.5 | 0.01 | 0.011 | 0.163 | 0.107 | 0.621 |
| Family | Veillonellaceae | 0.008 | 0.071 | 0.814 | 0.007 | 0.025 | 12.83 | 9.768 | 4.629 |
| Family | Bifidobacteriaceae | 0.02 | 0.112 | 0.708 | 0.018 | 0.063 | 0.778 | 1.57 | 0.001 |
| Family | Lachnospiraceae | 0.02 | 0.112 | 0.834 | 0.03 | 0.025 | 9.526 | 8.669 | 17.51 |
| Family | Clostridiaceae | 0.035 | 0.165 | 0.956 | 0.041 | 0.058 | 3.31 | 4.171 | 9.579 |
| Family | Enterobacteriaceae | 0.058 | 0.197 | 0.275 | 0.145 | 0.054 | 0.286 | 0.133 | 1.526 |
| Family | Enterococcaceae | 0.063 | 0.197 | 0.69 | 0.058 | 0.103 | 0.054 | 0.025 | 0.106 |
| Family | Porphyromonadaceae | 0.053 | 0.197 | 0.789 | 0.085 | 0.052 | 0.223 | 0.145 | 0.52 |
| Family | Lactobacillaceae | 0.077 | 0.216 | 0.651 | 0.074 | 0.186 | 8.919 | 5.226 | 0.718 |
| Family | Succinivibrionaceae | 0.151 | 0.384 | 0.718 | 0.135 | 0.233 | 6.977 | 8.86 | 2.369 |
| Family | Fusobacteriaceae | 0.172 | 0.4 | 0.939 | 0.215 | 0.208 | 2.378 | 1.874 | 5.947 |
| Family | Peptococcaceae | 0.271 | 0.571 | 0.455 | 0.396 | 0.328 | 0.233 | 0.17 | 0.636 |
| Family | Prevotellaceae | 0.292 | 0.571 | 0.388 | 0.504 | 0.351 | 28.688 | 32.972 | 26.134 |
| Family | Streptococcaceae | 0.306 | 0.571 | 0.582 | 0.413 | 0.593 | 1.247 | 0.701 | 0.526 |
| Family | Alcaligenaceae | 0.47 | 0.737 | 0.746 | 0.659 | 0.744 | 1.231 | 1.689 | 2.227 |
| Family | Ruminococcaceae | 0.474 | 0.737 | 0.719 | 0.666 | 0.689 | 3.049 | 3.664 | 4.127 |
| Family | Turicibacteraceae | 0.471 | 0.737 | 0.62 | 0.666 | 0.8 | 0.347 | 0.162 | 0.047 |
| Family | S24-7 | 0.579 | 0.853 | 0.73 | 1 | 0.819 | 0.163 | 0.22 | 0.219 |
| Family | Desulfovibrionaceae | 0.644 | 0.899 | 0.765 | 0.683 | 1 | 0.27 | 0.295 | 0.095 |
| Family | Erysipelotrichaceae | 0.706 | 0.899 | 0.845 | 1 | 0.879 | 4.539 | 3.491 | 2.686 |
| Family | Odoribacteraceae | 0.692 | 0.899 | 0.704 | 0.838 | 1 | 0.157 | 0.209 | 0.151 |
| Family | Coriobacteriaceae | 0.748 | 0.91 | 0.843 | 0.777 | 1 | 1.419 | 1.374 | 1.215 |
| Family | Helicobacteraceae | 0.806 | 0.926 | 1 | 0.967 | 0.912 | 0.326 | 0.227 | 0.593 |
| Family | Peptostreptococcaceae | 0.827 | 0.926 | 0.897 | 1 | 1 | 0.26 | 0.206 | 0.169 |
| Family | Paraprevotellaceae | 0.875 | 0.933 | 1 | 1 | 0.983 | 6.22 | 7.075 | 6.477 |
| Family | Rikenellaceae | 0.9 | 0.933 | 1 | 0.843 | 1 | 0.069 | 0.083 | 0.123 |
| Family | Campylobacteraceae | 0.971 | 0.971 | 0.938 | 1 | 1 | 0.129 | 0.151 | 0.119 |
| Genus | Bacteroides | 0.003 | 0.14 | 0.976 | 0.004 | 0.007 | 4.718 | 4.598 | 9.206 |
| Genus | Megasphaera | 0.012 | 0.144 | 0.198 | 0.009 | 0.226 | 7.901 | 2.9 | 0.079 |
| Genus | Ruminococcus | 0.013 | 0.144 | 0.99 | 0.016 | 0.025 | 1.438 | 1.661 | 3.469 |
| Genus | Slackia | 0.014 | 0.144 | 0.099 | 0.12 | 0.011 | 0.036 | 0.011 | 0.056 |
| Genus | Veillonella | 0.008 | 0.144 | 0.814 | 0.007 | 0.025 | 12.83 | 9.768 | 4.629 |
| Genus | Bifidobacterium | 0.02 | 0.171 | 0.708 | 0.018 | 0.063 | 0.778 | 1.57 | 0.001 |
| Genus | Bulleidia | 0.025 | 0.186 | 0.749 | 0.044 | 0.026 | 1.243 | 1.395 | 0.401 |
| Genus | Blautia | 0.045 | 0.207 | 0.212 | 0.15 | 0.039 | 3.373 | 2.256 | 6.054 |

| | | | | | | | | | |
|---|---|---|---|---|---|---|---|---|---|
| Genus | Catenibacterium | 0.043 | 0.207 | 0.57 | 0.038 | 0.147 | 1.362 | 0.841 | 0.415 |
| Genus | Clostridium | 0.035 | 0.207 | 0.984 | 0.044 | 0.052 | 3.275 | 4.075 | 9.517 |
| Genus | Escherichia | 0.037 | 0.207 | 0.253 | 0.105 | 0.034 | 0.283 | 0.128 | 1.521 |
| Genus | Parabacteroides | 0.053 | 0.226 | 0.789 | 0.085 | 0.052 | 0.223 | 0.144 | 0.52 |
| Genus | Enterococcus | 0.063 | 0.248 | 0.69 | 0.058 | 0.103 | 0.054 | 0.025 | 0.106 |
| Genus | Lactobacillus | 0.077 | 0.282 | 0.651 | 0.074 | 0.186 | 8.919 | 5.225 | 0.717 |
| Genus | Butyricicoccus | 0.09 | 0.286 | 0.938 | 0.12 | 0.101 | 0.175 | 0.209 | 0.299 |
| Genus | Mogibacterium | 0.087 | 0.286 | 0.277 | 0.107 | 0.506 | 0.003 | 0.006 | 0 |
| Genus | Faecalibacterium | 0.139 | 0.418 | 0.911 | 0.167 | 0.183 | 1.005 | 0.957 | 1.598 |
| Genus | Succinivibrio | 0.151 | 0.427 | 0.718 | 0.135 | 0.233 | 6.977 | 8.86 | 2.369 |
| Genus | Acidaminococcus | 0.193 | 0.492 | 0.665 | 0.212 | 0.357 | 0.04 | 0.066 | 0.002 |
| Genus | Dorea | 0.188 | 0.492 | 0.287 | 0.43 | 0.202 | 0.843 | 0.725 | 1.083 |
| Genus | Fusobacterium | 0.263 | 0.535 | 0.887 | 0.337 | 0.322 | 2.005 | 1.755 | 3.896 |
| Genus | Holdemania | 0.243 | 0.535 | 0.892 | 0.309 | 0.299 | 0.002 | 0.003 | 0.006 |
| Genus | Megamonas | 0.225 | 0.535 | 0.391 | 0.38 | 0.256 | 1.679 | 2.9 | 1.293 |
| Genus | Oribacterium | 0.271 | 0.535 | 0.324 | 0.557 | 0.352 | 0.064 | 0.1 | 0.03 |
| Genus | p-75-a5 | 0.273 | 0.535 | 0.406 | 0.447 | 0.33 | 0.002 | 0 | 0.005 |
| Genus | Peptococcus | 0.271 | 0.535 | 0.455 | 0.396 | 0.328 | 0.233 | 0.17 | 0.636 |
| Genus | Streptococcus | 0.306 | 0.577 | 0.582 | 0.413 | 0.593 | 1.247 | 0.701 | 0.526 |
| Genus | Porphyromonas | 0.344 | 0.627 | 0.561 | 0.436 | 0.679 | 0 | 0 | 0 |
| Genus | Butyrivibrio | 0.4 | 0.681 | 0.851 | 0.681 | 0.346 | 0.01 | 0.018 | 0.002 |
| Genus | Prevotella | 0.388 | 0.681 | 0.771 | 0.357 | 0.62 | 34.902 | 40.034 | 32.591 |
| Genus | Oscillospira | 0.441 | 0.703 | 0.486 | 0.83 | 0.931 | 0.527 | 0.641 | 0.75 |
| Genus | Roseburia | 0.438 | 0.703 | 0.652 | 0.48 | 0.644 | 0.309 | 0.308 | 0.477 |
| Genus | Sutterella | 0.47 | 0.707 | 0.746 | 0.659 | 0.744 | 1.231 | 1.688 | 2.227 |
| Genus | Turicibacter | 0.471 | 0.707 | 0.62 | 0.666 | 0.8 | 0.347 | 0.162 | 0.047 |
| Genus | Anaerobiospirillum | 0.502 | 0.732 | 0.874 | 0.613 | 0.844 | 2.813 | 1.776 | 1.65 |
| Genus | Peptostreptococcus | 0.527 | 0.747 | 0.685 | 0.571 | 0.781 | 0.003 | 0 | 0.004 |
| Genus | Candidatus Arthromitus | 0.594 | 0.819 | 0.773 | 0.606 | 1 | 0.032 | 0.086 | 0.006 |
| Genus | Desulfovibrio | 0.644 | 0.842 | 0.765 | 0.683 | 1 | 0.27 | 0.296 | 0.095 |
| Genus | Eubacterium | 0.627 | 0.842 | 0.767 | 0.646 | 1 | 1.768 | 1.173 | 1.455 |
| Genus | Alistipes | 0.711 | 0.863 | 1 | 0.818 | 0.914 | 0.01 | 0.013 | 0.019 |
| Genus | Dialister | 0.694 | 0.863 | 0.932 | 1 | 0.688 | 0.946 | 1.124 | 1.03 |
| Genus | Odoribacter | 0.692 | 0.863 | 0.704 | 0.838 | 1 | 0.157 | 0.209 | 0.151 |
| Genus | Adlercreutzia | 0.793 | 0.894 | 0.75 | 0.96 | 1 | 0.01 | 0.008 | 0.007 |
| Genus | Collinsella | 0.787 | 0.894 | 0.908 | 0.787 | 1 | 1.262 | 1.239 | 1.087 |
| Genus | Helicobacter | 0.806 | 0.894 | 1 | 0.967 | 0.912 | 0.326 | 0.227 | 0.593 |
| Genus | Phascolarctobacterium | 0.759 | 0.894 | 1 | 0.993 | 0.813 | 2.229 | 2.755 | 2.216 |
| Genus | Campylobacter | 0.831 | 0.901 | 0.927 | 1 | 0.995 | 0.454 | 0.377 | 0.711 |
| Genus | Paraprevotella | 0.875 | 0.93 | 1 | 1 | 0.983 | 6.22 | 7.075 | 6.477 |
| Genus | Coprococcus | 0.91 | 0.947 | 1 | 1 | 0.931 | 0.429 | 0.773 | 0.231 |
| Genus | Anaerotruncus | 0.961 | 0.981 | 1 | 0.913 | 1 | 0.003 | 0.003 | 0.003 |
| Genus | RFN20 | 0.987 | 0.987 | 1 | 0.976 | 1 | 0.004 | 0.005 | 0.003 |
| Species | gnavus | 0.002 | 0.061 | 0.451 | 0.004 | 0.004 | 0.7 | 0.355 | 2.699 |
| Species | hiranonis | 0.005 | 0.065 | 0.634 | 0.011 | 0.006 | 2.582 | 2.113 | 7.067 |
| Species | dolichum | 0.023 | 0.15 | 0.249 | 0.07 | 0.021 | 0.019 | 0.008 | 0.042 |
| Species | producta | 0.021 | 0.15 | 0.488 | 0.03 | 0.029 | 0.969 | 0.787 | 2.231 |
| Species | coli | 0.037 | 0.191 | 0.253 | 0.105 | 0.034 | 0.283 | 0.128 | 1.521 |
| Species | adolescentis | 0.053 | 0.197 | 0.301 | 0.048 | 0.357 | 0.651 | 1.519 | 0.001 |
| Species | cylindroides | 0.051 | 0.197 | 0.803 | 0.079 | 0.051 | 0.005 | 0.009 | 0 |
| Species | cecorum | 0.063 | 0.205 | 0.69 | 0.058 | 0.103 | 0.054 | 0.025 | 0.106 |

| | | | | | | | | | |
|---|---|---|---|---|---|---|---|---|---|
| Species | pullicaecorum | 0.09 | 0.259 | 0.938 | 0.12 | 0.101 | 0.175 | 0.209 | 0.299 |
| Species | p-1630-c5 | 0.104 | 0.272 | 0.932 | 0.124 | 0.14 | 0.568 | 0.554 | 0.275 |
| Species | torques | 0.117 | 0.277 | 0.233 | 0.334 | 0.115 | 0.005 | 0.002 | 0.006 |
| Species | prausnitzii | 0.148 | 0.321 | 0.861 | 0.172 | 0.208 | 0.978 | 0.938 | 1.564 |
| Species | uniformis | 0.233 | 0.465 | 0.606 | 0.263 | 0.459 | 0.073 | 0.045 | 0.103 |
| Species | ruminis | 0.254 | 0.472 | 0.613 | 0.275 | 0.345 | 0.488 | 0.915 | 0 |
| Species | luteciae | 0.306 | 0.53 | 0.582 | 0.413 | 0.593 | 1.206 | 0.682 | 0.516 |
| Species | copri | 0.334 | 0.542 | 0.416 | 0.543 | 0.416 | 28.452 | 32.7 | 26.01 |
| Species | aerofaciens | 0.367 | 0.561 | 0.555 | 0.553 | 0.711 | 0.574 | 0.544 | 0.224 |
| Species | coprophilus | 0.42 | 0.607 | 0.567 | 0.52 | 0.572 | 0.072 | 0.066 | 0.004 |
| Species | plebeius | 0.445 | 0.61 | 0.61 | 0.683 | 0.688 | 0.394 | 0.575 | 0.809 |
| Species | stercoris | 0.488 | 0.634 | 0.501 | 0.705 | 0.693 | 0.45 | 0.241 | 0.653 |
| Species | alactolyticus | 0.62 | 0.739 | 0.68 | 1 | 0.942 | 0.009 | 0.007 | 0.004 |
| Species | biforme | 0.647 | 0.739 | 0.744 | 0.701 | 1 | 1.744 | 1.156 | 1.412 |
| Species | citroniae | 0.682 | 0.739 | 0.843 | 0.686 | 1 | 0.01 | 0.008 | 0.011 |
| Species | massiliensis | 0.711 | 0.739 | 1 | 0.818 | 0.914 | 0.01 | 0.013 | 0.019 |
| Species | perfringens | 0.693 | 0.739 | 0.729 | 0.804 | 1 | 0.587 | 1.726 | 2.381 |
| Species | reuteri | 0.989 | 0.989 | 1 | 0.999 | 1 | 0.135 | 0.432 | 0.07 |

## OW cats

| | | | | P values (Dunn's test) | | | Mean | | | |
|---|---|---|---|---|---|---|---|---|---|---|
| Rank | Taxanomy | P_KW* | FDR | T1_CON vs T2_CON | T1_CON vs T2_HPLC | T2_CON vs T2_HPLC | T1_CON | T2_CON | T2_HPLC | FC** |
| Phy | Fusobacteria | 0 | 0.001 | 0.651 | 0 | 0 | 0.669 | 0.477 | 3.882 | 8.1 |
| Phy | Actinobacteria | 0.18 | 0.311 | 0.86 | 0.28 | 0.162 | 6.497 | 5.109 | 1.981 | -2.6 |
| Phy | Bacteroidetes | 0.187 | 0.311 | 0.667 | 0.205 | 0.342 | 36.365 | 37.602 | 43.95 | 1.2 |
| Phy | Firmicutes | 0.771 | 0.771 | 0.766 | 1 | 1 | 47.836 | 47.58 | 44.286 | -1.1 |
| Phy | Proteobacteria | 0.682 | 0.771 | 0.991 | 1 | 0.719 | 8.472 | 9.049 | 5.722 | -1.6 |
| Fam | Fusobacteriaceae | 0 | 0.004 | 0.651 | 0 | 0 | 0.669 | 0.477 | 3.882 | 8.1 |
| Fam | Veillonellaceae | 0 | 0.004 | 0.691 | 0 | 0.003 | 19.633 | 16.554 | 4.859 | -3.4 |
| Fam | Bifidobacteriaceae | 0.002 | 0.018 | 0.527 | 0.003 | 0.004 | 2.54 | 2.133 | 0.003 | -711 |
| Fam | Clostridiaceae | 0.007 | 0.036 | 0.851 | 0.007 | 0.02 | 3.041 | 3.061 | 6.925 | 2.3 |
| Fam | Lachnospiraceae | 0.007 | 0.036 | 0.31 | 0.005 | 0.099 | 9.471 | 11.309 | 15.852 | 1.4 |
| Fam | Ruminococcaceae | 0.008 | 0.036 | 0.174 | 0.006 | 0.196 | 3.788 | 5.12 | 7.037 | 1.4 |
| Fam | Mogibacteriaceae | 0.011 | 0.037 | 0.048 | 0.02 | 0.619 | 0.158 | 0.317 | 0.586 | 1.8 |
| Fam | Peptococcaceae | 0.01 | 0.037 | 0.171 | 0.009 | 0.228 | 0.267 | 0.407 | 0.669 | 1.6 |
| Fam | Porphyromonadaceae | 0.012 | 0.038 | 0.013 | 0.877 | 0.029 | 0.14 | 0.601 | 0.171 | -3.5 |
| Fam | Rikenellaceae | 0.015 | 0.041 | 0.022 | 0.505 | 0.021 | 0.048 | 0.114 | 0.014 | -8.1 |
| Fam | Alcaligenaceae | 0.031 | 0.079 | 0.817 | 0.05 | 0.034 | 0.859 | 0.772 | 2.24 | 2.9 |
| Fam | Odoribacteraceae | 0.038 | 0.088 | 0.045 | 0.965 | 0.058 | 0.199 | 0.534 | 0.153 | -3.5 |
| Fam | S24-7 | 0.105 | 0.225 | 0.294 | 0.13 | 0.528 | 0.135 | 0.195 | 0.245 | 1.3 |
| Fam | Bacteroidaceae | 0.124 | 0.248 | 0.195 | 0.216 | 0.804 | 4.002 | 6.046 | 5.884 | -1 |
| Fam | Peptostreptococcaceae | 0.141 | 0.263 | 0.144 | 0.472 | 0.413 | 0.09 | 0.175 | 0.096 | -1.8 |
| Fam | Lactobacillaceae | 0.153 | 0.268 | 0.261 | 0.231 | 0.804 | 1.886 | 1.747 | 1.358 | -1.3 |

| | | | | | | | | | | |
|---|---|---|---|---|---|---|---|---|---|---|
| Fam | Erysipelotrichaceae | 0.188 | 0.31 | 0.289 | 0.44 | 0.217 | 4.356 | 5.721 | 3.741 | -1.5 |
| Fam | Succinivibrionaceae | 0.227 | 0.353 | 0.912 | 0.293 | 0.271 | 6.556 | 7.373 | 2.579 | -2.9 |
| Fam | Coriobacteriaceae | 0.281 | 0.393 | 0.947 | 0.383 | 0.31 | 3.956 | 2.973 | 1.975 | -1.5 |
| Fam | Prevotellaceae | 0.271 | 0.393 | 0.434 | 0.427 | 0.324 | 27.029 | 23.426 | 30.993 | 1.3 |
| Fam | Enterococcaceae | 0.297 | 0.396 | 0.618 | 0.331 | 0.406 | 0.527 | 0.041 | 0.127 | 3.1 |
| Fam | Paraprevotellaceae | 0.417 | 0.531 | 0.611 | 0.636 | 0.716 | 4.677 | 6.401 | 6.327 | -1 |
| Fam | Turicibacteraceae | 0.478 | 0.582 | 0.505 | 0.693 | 0.675 | 0.249 | 0.195 | 0.058 | -3.4 |
| Fam | Enterobacteriaceae | 0.507 | 0.592 | 0.879 | 0.903 | 0.464 | 0.204 | 0.037 | 0.277 | 7.5 |
| Fam | Campylobacteraceae | 0.546 | 0.612 | 0.959 | 0.659 | 0.848 | 0.259 | 0.207 | 0.153 | -1.4 |
| Fam | Desulfovibrionaceae | 0.632 | 0.632 | 1 | 0.826 | 0.789 | 0.158 | 0.294 | 0.151 | -1.9 |
| Fam | Helicobacteraceae | 0.629 | 0.632 | 0.779 | 0.657 | 1 | 0.418 | 0.338 | 0.164 | -2.1 |
| Fam | Streptococcaceae | 0.604 | 0.632 | 0.993 | 0.93 | 0.666 | 3.476 | 1.716 | 1.739 | 1 |
| Gen | Faecalibacterium | 0 | 0.002 | 0.868 | 0 | 0.001 | 0.714 | 0.717 | 3.211 | 4.5 |
| Gen | Megasphaera | 0 | 0.002 | 0.851 | 0 | 0.001 | 12.379 | 10.344 | 0.124 | -83.4 |
| Gen | Fusobacterium | 0 | 0.003 | 0.651 | 0 | 0 | 0.659 | 0.468 | 3.75 | 8 |
| Gen | Veillonella | 0 | 0.004 | 0.691 | 0 | 0.003 | 19.633 | 16.554 | 4.859 | -3.4 |
| Gen | Bifidobacterium | 0.002 | 0.019 | 0.527 | 0.003 | 0.004 | 2.54 | 2.133 | 0.003 | -711 |
| Gen | Clostridium | 0.007 | 0.057 | 0.8 | 0.007 | 0.023 | 3.014 | 3.053 | 6.862 | 2.2 |
| Gen | Blautia | 0.01 | 0.064 | 0.224 | 0.007 | 0.175 | 3.246 | 3.467 | 5.52 | 1.6 |
| Gen | Peptococcus | 0.01 | 0.064 | 0.171 | 0.009 | 0.228 | 0.267 | 0.407 | 0.669 | 1.6 |
| Gen | Alistipes | 0.018 | 0.089 | 0.035 | 0.704 | 0.018 | 0.015 | 0.031 | 0.007 | -4.4 |
| Gen | Anaerotruncus | 0.017 | 0.089 | 0.137 | 0.107 | 0.013 | 0.003 | 0.007 | 0 | na |
| Gen | Parabacteroides | 0.019 | 0.089 | 0.023 | 0.947 | 0.036 | 0.137 | 0.491 | 0.157 | -3.1 |
| Gen | Sutterella | 0.031 | 0.132 | 0.817 | 0.05 | 0.034 | 0.858 | 0.772 | 2.24 | 2.9 |
| Gen | Odoribacter | 0.038 | 0.147 | 0.045 | 0.965 | 0.058 | 0.199 | 0.534 | 0.153 | -3.5 |
| Gen | Butyricicoccus | 0.049 | 0.166 | 0.168 | 0.065 | 0.54 | 0.191 | 0.28 | 0.328 | 1.2 |
| Gen | p-75-a5 | 0.047 | 0.166 | 0.315 | 0.046 | 0.311 | 0.002 | 0.005 | 0.008 | 1.6 |
| Gen | Catenibacterium | 0.063 | 0.192 | 0.231 | 0.196 | 0.056 | 1.127 | 1.873 | 0.475 | -3.9 |
| Gen | Roseburia | 0.064 | 0.192 | 0.075 | 0.921 | 0.095 | 0.416 | 0.96 | 0.244 | -3.9 |
| Gen | Megamonas | 0.071 | 0.201 | 0.377 | 0.065 | 0.331 | 2.256 | 1.592 | 0.486 | -3.3 |
| Gen | Ruminococcus | 0.08 | 0.216 | 0.529 | 0.075 | 0.244 | 1.417 | 1.651 | 2.144 | 1.3 |
| Gen | Slackia | 0.096 | 0.245 | 0.091 | 0.453 | 0.331 | 0.036 | 0.086 | 0.08 | -1.1 |
| Gen | Bacteroides | 0.124 | 0.3 | 0.195 | 0.216 | 0.804 | 3.997 | 6.04 | 5.871 | -1 |
| Gen | RFN20 | 0.129 | 0.3 | 0.439 | 0.13 | 0.422 | 0.001 | 0.003 | 0.006 | 2 |
| Gen | Bulleidia | 0.144 | 0.32 | 0.974 | 0.186 | 0.169 | 2.215 | 2.517 | 1.276 | -2 |
| Gen | Lactobacillus | 0.153 | 0.322 | 0.261 | 0.231 | 0.804 | 1.886 | 1.747 | 1.358 | -1.3 |
| Gen | Oscillospira | 0.158 | 0.322 | 0.187 | 0.328 | 0.954 | 0.612 | 0.944 | 0.917 | -1 |
| Gen | Adlercreutzia | 0.232 | 0.378 | 0.411 | 0.327 | 0.66 | 0.009 | 0.013 | 0.013 | -1 |
| Gen | Coprococcus | 0.218 | 0.378 | 0.3 | 0.366 | 0.818 | 0.45 | 0.478 | 0.437 | -1.1 |
| Gen | Dorea | 0.228 | 0.378 | 0.335 | 0.376 | 0.702 | 0.721 | 1.056 | 0.768 | -1.4 |
| Gen | Eubacterium | 0.245 | 0.378 | 0.492 | 0.267 | 0.969 | 0.948 | 1.266 | 1.48 | 1.2 |
| Gen | Mogibacterium | 0.211 | 0.378 | 0.643 | 0.235 | 0.392 | 0.008 | 0.005 | 0.001 | -5 |
| Gen | Porphyromonas | 0.24 | 0.378 | 0.321 | 0.465 | 0.605 | 0.002 | 0.109 | 0.014 | -7.8 |

| | | | | | | | | | | |
|---|---|---|---|---|---|---|---|---|---|---|
| Gen | Prevotella | 0.241 | 0.378 | 0.522 | 0.312 | 0.3 | 31.696 | 29.813 | 37.31 | 1.3 |
| Gen | Succinivibrio | 0.227 | 0.378 | 0.912 | 0.293 | 0.271 | 6.556 | 7.373 | 2.579 | -2.9 |
| Gen | Oribacterium | 0.253 | 0.38 | 0.626 | 0.275 | 0.346 | 0.123 | 0.099 | 0.191 | 1.9 |
| Gen | Phascolarctobacterium | 0.278 | 0.405 | 0.65 | 0.293 | 0.389 | 1.35 | 1.012 | 2.342 | 2.3 |
| Gen | Enterococcus | 0.297 | 0.421 | 0.618 | 0.331 | 0.406 | 0.527 | 0.041 | 0.127 | 3.1 |
| Gen | Peptostreptococcus | 0.351 | 0.484 | 0.5 | 0.951 | 0.379 | 0.001 | 0.002 | 0.004 | 2 |
| Gen | Acidaminococcus | 0.418 | 0.547 | 0.587 | 0.565 | 0.755 | 0.841 | 0.404 | 0.014 | -28.9 |
| Gen | Paraprevotella | 0.417 | 0.547 | 0.611 | 0.636 | 0.716 | 4.677 | 6.401 | 6.327 | -1 |
| Gen | Dialister | 0.474 | 0.595 | 0.728 | 0.484 | 0.742 | 2.595 | 2.962 | 1.881 | -1.6 |
| Gen | Turicibacter | 0.478 | 0.595 | 0.505 | 0.693 | 0.675 | 0.249 | 0.195 | 0.058 | -3.4 |
| Gen | Collinsella | 0.495 | 0.601 | 0.494 | 0.74 | 0.707 | 2.104 | 2.132 | 1.762 | -1.2 |
| Gen | Holdemania | 0.556 | 0.66 | 0.992 | 0.659 | 0.863 | 0.003 | 0.004 | 0.004 | -1 |
| Gen | Candidatus Arthromitus | 0.598 | 0.671 | 0.769 | 0.621 | 1 | 0.024 | 0.002 | 0.018 | 9 |
| Gen | Desulfovibrio | 0.632 | 0.671 | 1 | 0.826 | 0.789 | 0.158 | 0.294 | 0.151 | -1.9 |
| Gen | Escherichia | 0.615 | 0.671 | 0.648 | 0.765 | 1 | 0.202 | 0.037 | 0.275 | 7.4 |
| Gen | Helicobacter | 0.629 | 0.671 | 0.779 | 0.657 | 1 | 0.418 | 0.338 | 0.164 | -2.1 |
| Gen | Streptococcus | 0.604 | 0.671 | 0.993 | 0.93 | 0.666 | 3.476 | 1.716 | 1.739 | 1 |
| Gen | Campylobacter | 0.698 | 0.726 | 0.74 | 0.808 | 1 | 0.677 | 0.545 | 0.317 | -1.7 |
| Gen | Butyrivibrio | 0.805 | 0.821 | 0.896 | 0.846 | 1 | 0.04 | 0.049 | 0.053 | 1.1 |
| Gen | Anaerobiospirillum | 0.836 | 0.836 | 1 | 0.975 | 0.954 | 1.347 | 1.627 | 1.331 | -1.2 |
| Spe | prausnitzii | 0 | 0.002 | 0.868 | 0 | 0.001 | 0.696 | 0.699 | 3.139 | 4.5 |
| Spe | gnavus | 0 | 0.004 | 0.974 | 0 | 0.001 | 0.339 | 0.317 | 1.6 | 5 |
| Spe | cylindroides | 0.001 | 0.011 | 0.892 | 0.002 | 0.003 | 0.035 | 0.022 | 0 | na |
| Spe | dolichum | 0.003 | 0.022 | 0.091 | 0.043 | 0.002 | 0.004 | 0 | 0.043 | na |
| Spe | ruminis | 0.006 | 0.031 | 0.374 | 0.004 | 0.068 | 0.214 | 0.215 | 0 | na |
| Spe | plebeius | 0.01 | 0.042 | 0.596 | 0.008 | 0.048 | 0.838 | 0.713 | 0.279 | -2.6 |
| Spe | hiranonis | 0.012 | 0.043 | 0.487 | 0.009 | 0.073 | 2.522 | 2.948 | 6.082 | 2.1 |
| Spe | massiliensis | 0.018 | 0.059 | 0.035 | 0.704 | 0.018 | 0.015 | 0.031 | 0.007 | -4.4 |
| Spe | adolescentis | 0.034 | 0.097 | 0.846 | 0.051 | 0.038 | 2.062 | 1.891 | 0.001 | -1891 |
| Spe | producta | 0.046 | 0.115 | 0.265 | 0.041 | 0.365 | 0.764 | 0.91 | 1.269 | 1.4 |
| Spe | pullicaecorum | 0.049 | 0.115 | 0.168 | 0.065 | 0.54 | 0.191 | 0.28 | 0.328 | 1.2 |
| Spe | torques | 0.059 | 0.129 | 0.319 | 0.061 | 0.351 | 0.001 | 0.001 | 0.003 | 3 |
| Spe | perfringens | 0.086 | 0.172 | 0.51 | 0.114 | 0.105 | 0.1 | 0.031 | 0.704 | 22.7 |
| Spe | aerofaciens | 0.146 | 0.259 | 0.757 | 0.127 | 0.235 | 0.813 | 0.653 | 0.384 | -1.7 |
| Spe | citroniae | 0.149 | 0.259 | 0.479 | 0.154 | 0.424 | 0.005 | 0.006 | 0.01 | 1.7 |
| Spe | reuteri | 0.172 | 0.28 | 0.844 | 0.204 | 0.238 | 0.004 | 0.012 | 0.039 | 3.3 |
| Spe | stercoris | 0.224 | 0.343 | 0.39 | 0.321 | 0.674 | 0.346 | 0.573 | 1.115 | 1.9 |
| Spe | biforme | 0.256 | 0.35 | 0.502 | 0.283 | 0.954 | 0.907 | 1.243 | 1.437 | 1.2 |
| Spe | copri | 0.253 | 0.35 | 0.39 | 0.44 | 0.3 | 26.581 | 23.107 | 30.811 | 1.3 |
| Spe | cecorum | 0.297 | 0.386 | 0.618 | 0.331 | 0.406 | 0.527 | 0.041 | 0.127 | 3.1 |
| Spe | uniformis | 0.333 | 0.412 | 0.398 | 0.579 | 0.414 | 0.116 | 0.175 | 0.062 | -2.8 |
| Spe | luteciae | 0.475 | 0.561 | 0.725 | 0.947 | 0.508 | 3.421 | 1.673 | 1.713 | 1 |
| Spe | p-1630-c5 | 0.528 | 0.597 | 0.699 | 0.574 | 0.827 | 0.995 | 1.182 | 0.616 | -1.9 |

| | | | | | | | | | | |
|---|---|---|---|---|---|---|---|---|---|---|
| Spe | E. coli | 0.615 | 0.666 | 0.648 | 0.765 | 1 | 0.202 | 0.037 | 0.275 | 7.4 |
| Spe | alactolyticus | 0.934 | 0.936 | 1 | 0.962 | 1 | 0.017 | 0.023 | 0.01 | -2.3 |
| Spe | coprophilus | 0.936 | 0.936 | 0.879 | 1 | 1 | 0.014 | 0.015 | 0.032 | 2.1 |

*KW, Kruskall-Wallis

**FC, fold change HPLC/CON

**Table S5.** LEfSe analysis on bacterial genus between OW-HPLC vs. OW-CON cats using the selection criteria of FDR < 0.05 and $\log_{10} LDA$ > 3.0.

| | P | FDR | HPLC | CON | LDAscore |
|---|---|---|---|---|---|
| g__Megasphaera | 0.00015705 | 0.0092881 | 12437 | 1036000 | 5.71 |
| g__Faecalibacterium | 0.00050654 | 0.0092881 | 321370 | 71700 | -5.1 |
| g__Fusobacterium | 0.00050654 | 0.0092881 | 375660 | 46866 | -5.22 |
| g__Bifidobacterium | 0.00060906 | 0.0092881 | 252.61 | 213520 | 5.03 |
| g__Anaerotruncus | 0.0051753 | 0.063139 | 0 | 699.42 | 2.54 |
| g__Alistipes | 0.0098836 | 0.10048 | 740.45 | 3014.4 | 3.06 |
| g__Catenibacterium | 0.015564 | 0.11868 | 47479 | 187530 | 4.85 |
| g__Clostridium | 0.015564 | 0.11868 | 687030 | 305690 | -5.28 |
| g__Odoribacter | 0.023342 | 0.14239 | 15327 | 52784 | 4.27 |
| g__Sutterella | 0.023342 | 0.14239 | 224260 | 77219 | -4.87 |
| g__Parabacteroides | 0.028366 | 0.14345 | 15723 | 47572 | 4.2 |
| g__Roseburia | 0.028366 | 0.14345 | 24506 | 96122 | 4.55 |
| g__Butyricimonas | 0.03057 | 0.14345 | 0 | 724.78 | 2.56 |
| g__Succinivibrio | 0.049281 | 0.21472 | 76627 | 515780 | 5.34 |
| g__Blautia | 0.058782 | 0.23905 | 553120 | 347170 | -5.01 |
| g__Prevotella | 0.11241 | 0.40336 | 3737500 | 2985600 | -5.58 |
| g__Desulfovibrio | 0.13638 | 0.41128 | 7047.4 | 27159 | 4 |
| g__Mitsuokella | 0.14679 | 0.41128 | 0 | 17759 | 3.95 |
| g__Peptococcus | 0.15093 | 0.41128 | 67031 | 40740 | -4.12 |
| g__Bulleidia | 0.15093 | 0.41128 | 127920 | 252140 | 4.79 |
| g__Actinomyces | 0.15585 | 0.41128 | 304.15 | 92.245 | -2.03 |
| g__Oribacterium | 0.16039 | 0.41128 | 19140 | 9898.2 | -3.66 |
| g__Phascolarctobacterium | 0.16181 | 0.41128 | 234820 | 101430 | -4.82 |
| g__Ruminococcus | 0.17362 | 0.42363 | 214210 | 165240 | -4.39 |
| g__Megamonas | 0.19809 | 0.46475 | 48674 | 159120 | 4.74 |
| g__Enterococcus | 0.21145 | 0.47638 | 12468 | 4092.3 | -3.62 |
| g__Mogibacterium | 0.22569 | 0.47638 | 147.47 | 463.31 | 2.2 |
| g__Collinsella | 0.22648 | 0.47638 | 176850 | 213450 | 4.26 |
| g__Turicibacter | 0.23452 | 0.47686 | 5824.5 | 19485 | 3.83 |
| g__Candidatus_Arthromitus | 0.28206 | 0.54255 | 1829.2 | 235.85 | -2.9 |
| g__Dialister | 0.28992 | 0.54255 | 188150 | 296410 | 4.73 |
| g__RFN20 | 0.29351 | 0.54255 | 589.1 | 259.54 | -2.22 |
| g__Slackia | 0.36435 | 0.65094 | 8036.4 | 8586 | 2.44 |
| g__Peptostreptococcus | 0.37508 | 0.65094 | 355.3 | 139.52 | -2.04 |
| g__Paraprevotella | 0.39555 | 0.65094 | 1032.1 | 1482.1 | 2.35 |
| g__Helicobacter | 0.39555 | 0.65094 | 16430 | 33781 | 3.94 |
| g__Streptococcus | 0.4055 | 0.65094 | 173920 | 171900 | -3.01 |
| g__Porphyromonas | 0.42805 | 0.66056 | 1344 | 10959 | 3.68 |
| g__Acidaminococcus | 0.44625 | 0.66056 | 1400.5 | 40494 | 4.29 |

| | | | | | |
|---|---|---|---|---|---|
| g__p_75_a5 | 0.45812 | 0.66056 | 768.27 | 503.54 | -2.13 |
| g__Gallibacterium | 0.46564 | 0.66056 | 43.691 | 320.6 | 2.14 |
| g__Epulopiscium | 0.46564 | 0.66056 | 9310.5 | 34.974 | -3.67 |
| g__Escherichia | 0.49302 | 0.68351 | 27496 | 3701.9 | -4.08 |
| g__Butyricicoccus | 0.54535 | 0.73925 | 32923 | 28020 | -3.39 |
| g__Butyrivibrio | 0.59391 | 0.77444 | 5280.9 | 4935.2 | -2.24 |
| g__Adlercreutzia | 0.5967 | 0.77444 | 1349.2 | 1338.3 | -0.81 |
| g__Veillonella | 0.62669 | 0.77763 | 58.88 | 56.687 | -0.322 |
| g__Sarcina | 0.62669 | 0.77763 | 40.914 | 21.616 | -1.03 |
| g__Lactobacillus | 0.65002 | 0.77763 | 135540 | 174520 | 4.29 |
| g__Dorea | 0.65015 | 0.77763 | 76920 | 105690 | 4.16 |
| g__Campylobacter | 0.76237 | 0.87744 | 15267 | 20701 | 3.43 |
| g__Holdemania | 0.77741 | 0.87818 | 321.46 | 387.75 | 1.53 |
| g__SMB53 | 0.81577 | 0.87818 | 656.71 | 188.22 | -2.37 |
| g__Oscillospira | 0.8206 | 0.87818 | 91899 | 92015 | 1.77 |
| g__Bacteroides | 0.8206 | 0.87818 | 588570 | 604700 | 3.91 |
| g__Eubacterium | 0.87983 | 0.92534 | 148220 | 126630 | -4.03 |
| g__Coprococcus | 0.93974 | 0.95787 | 43826 | 47816 | 3.3 |
| g__Delftia | 0.94217 | 0.95787 | 17.192 | 18.32 | 0.194 |
| g__Anaerobiospirillum | 0.96984 | 0.96984 | 133230 | 162920 | 4.17 |

**Table S6.** PICRUSt-predicted KEGG metagenomic orthologous functions between OW-HPLC and OW-CON cats. KO pathways were selected using LEfSe with the selection criteria of P < 0.05 and $\log_{10} LDA$ > 2.5.

| KEGG pathway | Class | log_LDA | P |
|---|---|---|---|
| One carbon pool by folate | CON | 2.7 | 0.019 |
| Peptidases | CON | 2.7 | 0.007 |
| Propanoate metabolism | CON | 2.7 | 0.007 |
| Fatty acid metabolism | CON | 2.6 | 0.049 |
| Tryptophan metabolism | CON | 2.6 | 0.028 |
| Glycolysis & Gluconeogenesis | CON | 2.6 | 0.002 |
| Pyruvate metabolism | CON | 2.6 | 0.01 |
| Lysine degradation | CON | 2.5 | 0.003 |
| TCA cycle | CON | 2.5 | 0.034 |
| Folate biosynthesis | CON | 2.5 | 0.023 |
